# Effects of measurement errors on relationships between resting-state functional connectivity and psychological phenotypes

**DOI:** 10.1038/s41598-025-13105-0

**Published:** 2025-08-19

**Authors:** Tomosumi Haitani, Yuki Sakai, Saori C. Tanaka

**Affiliations:** 1 ATR Brain Information Communication Research Laboratory Group, 2-2-2 Hikaridai Seika-Cho, Soraku-Gun, Kyoto, 619-0288 Japan; 2 Division of Information Science, Graduate School of Science and Technology, Nara Institute of Science and Technology, 8916-5 Takayama-Cho, Ikoma, Nara 630-0192 Japan

**Keywords:** Resting-state functional connectivity, Structural equation modeling, Measurement error, Human connectome project, Prediction, Neuroscience, Psychology

## Abstract

Recent neuroscientific studies have focused on interindividual relationships between resting-state functional connectivity (RSFC) and psychological phenotypes using large datasets with repeated measurements, including the Human Connectome Project (HCP). However, previous studies on RSFC-phenotype relationships have failed to differentiate trait, state, and error effects of RSFC. Latent functional connectivity, which can be estimated in structural equation model (SEM), can be useful in finding RSFC-phenotype relationships controlling state and error effects. We also accounted for measurement errors in psychological phenotypes at the test-, subscale-, or item-level. This study investigates: (i) how measurement errors, including state effects, weaken the associations between RSFC and psychological phenotypes, including cognition, mental health, and personality, and influence sample size planning and (ii) predictive accuracy on the phenotypes from RSFC, using SEM. We found that the extent of the weakening of RSFC-phenotype associations ranged from 15.3 to 33.8% across the phenotypes, and they were higher in sensorimotor networks than in higher order cognitive networks. Importantly, measurement errors can lead to requirement of about double sample size to find RSFC-phenotype associations in general. Factor scores of RSFC enhanced the coefficients of determination under some conditions. Future studies should explore more effective predictive methods by accounting for measurement errors.

**Supplementary Information:**

The online version contains supplementary material available at 10.1038/s41598-025-13105-0.

## Introduction

Recent neuroscientific studies have focused on identifying interindividual relationships between brain measures and psychological phenotypes^1–3^. Resting-state functional connectivity (RSFC) is one of the most used measures to study interindividual differences. In the context of analyses of RSFC, nodes represent brain regions while edges represent the functional relationships between node signals, often defined as Pearson’s correlations. Previous studies have reported that RSFC is associated with various psychological phenotypes, including cognition, psychopathology, and personality^4^. However, RSFC exhibits day-to-day and scan-to-scan variability^5^with poor test-retest reliability^6^. Prior studies have shown that within-individual variability of RSFC accounts for a significant proportion of variability of RSFC, and may prevent the identification of stable biomarkers of mental disorders^7^; thus, correctly capturing within-individual variability through repeated measurements is essential to advance not only studies on RSFC-phenotype relationships, but also clinical applications of RSFC. In addition to RSFC, psychological phenotypes are measured with errors and previous studies have shown that the reliability of psychological phenotypes can also significantly impact RSFC-phenotype relationships^8,9^. Thus, measurement errors should be considered when investigating the relationships between RSFC and psychological phenotypes^10^.

In psychometrics, measurement error consists of three types: transient error (also referred to as state effects or intraindividual variability), specific factor error or method effects, and random error^11,12^. Transient errors represent random state variations of participants in responses to measures across time. Previous studies using large datasets have shown that state effects of RSFC can be particularly pronounced in sensorimotor networks^7,13–15^. Specific factor errors are systematic errors inherent to the measure and unrelated to the construct of interest. For example, order of scans may lead to specific factor error of RSFC^13^ and individual difference of interpretation of instructions or wording of items leads to test- or item-level specific factor error of psychological phenotypes^12^. Random errors are caused by variations within a given time point, such as momentary variation of attention in taking cognitive tests or answering questionnaire items^12^. It is shown that test-retest reliability, which assesses the degrees of transient and random response errors, of phenotypes attenuates RSFC-phenotype relationships^8^. However, effects of test-, subscale-, or item-level measurement errors, including specific factor and random response errors, of psychological phenotypes on RSFC-phenotype relationships have largely been overlooked. Considering measurement errors of phenotypes can lead to more efficient estimates of them. For example, general cognitive ability can be efficiently estimated considering specific factor and random errors of domain-specific cognitive abilities, including language, executive functioning, and memory^16^. It is possible that state, method, and random error effects of both RSFC and phenotypes obscure their true relationships.

Recent neuroscientific studies have increasingly incorporated psychometrics and latent variable models into studies on brain-phenotype relationships^1,17–19^. Structural equation modeling (SEM) is a latent variable modeling technique frequently used in psychometrics. Typically, SEM consists of two components, namely measurement and structural models. Among several SEM approaches, covariance-based SEM (CB-SEM) with reflective measurement models have been used in previous studies of RSFC-phenotype relationship^17,18^. Reflective measurement models allow us to examine measurement error by assuming that the variances and covariances of indicators (observed variables such as test, subscale, or item scores) are explained by a common latent factor and measurement error. Confirmatory factor analysis (CFA) is often used to establish reflective measurement models. The structural model in CB-SEM can then reveal associations while correcting for measurement error attenuation. Teeuw et al.^18^demonstrated that CB-SEM increased the strength of RSFC-phenotype associations by 1.2-fold on average by correcting for measurement error attenuation in RSFC, using the Human Connectome Project (HCP) dataset^20^. Specifically, the resting-state functional magnetic resonance imaging (fMRI) data in the HCP were collected across four runs over two days^20^and reflective measurement models were applied to edges derived from independent component analysis, by splitting the RSFC data into two halves (day 1 and 2). However, measurement models were not applied to phenotype measures, as only their average scores were used.

SEM or CFA can be useful for not only examining RSFC-phenotype associations but also for RSFC-phenotype prediction^19,21–23^. While previous studies on RSFC-phenotype predictions have explored the accuracy of predictive models, including regularized regressions^24^measurement error can substantially impact the prediction accuracy of these models^22^. Factor scores, which serve as approximations of unobserved latent factors, can be computed for subsequent analyses after the CFA^25^. Factor score estimates of latent functional connectivity (FC) across different tasks, including resting conditions, have been shown to provide better predictive accuracy for general intelligence compared to average scores of observed RSFC^19^. Although recent psychometric studies have empirically explored the utility of both average (or sum) and factor scores^23,26,27^whether factor scores provide better predictive accuracy than sum or average scores, specifically in the context of RSFC-phenotype prediction, remains unclear. Another CB-SEM-based prediction method is operative prediction, where scores of observed exogenous (response) variable(s) are predicted from those of observed endogenous (predictor) variables based on estimated joint distributions of the variables^21,28^. CB-SEM-based operative prediction can outperform regularized linear regressions, including elastic net regressions, particularly in relatively small samples (*N* ≤ 1,000)^21^.

**Fig. 1:**
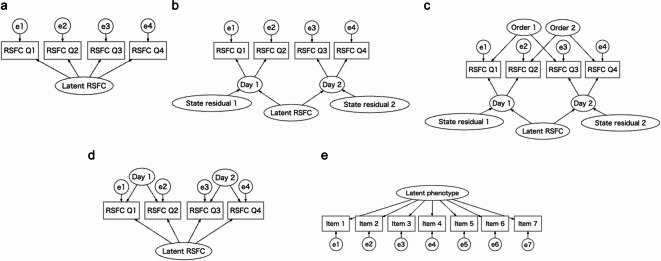
Measurement models used in the present study. (**a**) Single-trait model. (**b**) Multistate single-trait model. **c** Multistate single-trait model with method factors of orders of measurements in days. (**d**) Bi-factor model. (**e**) An example of a phenotype measurement model including seven items. RSFC Q1, Q2, Q3, and Q4 represent RSFCs measured with the right-left phase encoding direction in day 1, left-right phase encoding direction in day 1, left-right phase encoding direction in day 2, and right-left phase encoding direction in day 2. Variances of the latent variables were standardized to 1 in (**a**–**e**), while factor loadings were equally constrained in the same types of factors (Latent RSFC, Day, and Order) in (**a**–**d**). Variances of exogenous latent variables are omitted.

The current study mainly builds upon the findings of Teeuw et al.^18^ by applying CB-SEM to both RSFC and phenotype measures, explicitly considering the effects of state and method on RSFC, as well as brain networks^29^. In the present study, we compared several types of models, including a baseline single-trait model, which enables the separation of trait and error effects through a single common latent factor (Fig. 1a); and a multistate single-trait model, a form of latent state-trait model which enables the separation of trait, state, and error effects through state (day) factors and a higher-order trait factor (Fig. 1b)^30^. We also focused on a multistate single-trait model with method factors, representing the measurement order in days (Fig. 1c)^30^. Multistate-single trait models enable variance decomposition of indicators^30^. Bi-factor models, in which a first-order latent common factor and orthogonal state factors are assumed (Fig. 1d), represent a possible alternative for multistate single-trait models^31^. Bi-factor models have previously been used in intra-class effect decomposition (ICED)^31^which also enables variance decomposition. We newly introduced latent state-trait modeling in neuroscience, which explicitly focuses on state effects, and could contribute to controlling within-individual variability. Measurement errors in phenotypes were also addressed by extracting a latent common factor within reflective measurement models. In this study, we assume unidimensionality and congenericity in phenotype measures; that is, covariances between indicators (e.g., test, subscale, or item scores) are explained by a single latent factor (unidimensionality), and factor loadings are allowed to vary across indicators (congenericity). Moreover, we examined the predictive accuracy of models using factor score estimates of latent RSFC as predictors and those of phenotypes as response variables, and operative prediction using item or subscale scores as response variables. Throughout these investigations, we considered the effects of global signal regression (GSR), as it can influence measurement error in both RSFC and RSFC-phenotype relationships^2,6,32^. We focused on edge-level RSFC and its relationships with cognition, mental health, and personality, as these phenotypes are frequently used in studies of RSFC-phenotype relationships^3,4^.

## Results

All SEM, including the measurement models, were fit using *lavaan* library^33^ in R, as described in the Methods section. Multivariate normality of the observed variables was assumed in SEM. Under non-normal data, parameter estimates are relatively accurate, while model fit statistics, including the chi-square statistics described below, are biased^34^.

Fits of SEM were predominantly evaluated through chi-square statistics, whose higher values represent the worse model fits. Model fits can be compared through differences in the chi-square (*Δχ*^2^)values of the nested models. When comparing between single-trait and multistate single trait models, the difference in the degrees of freedom was 1 (5 − 4) and upper-tail critical value of chi-square distribution (with *p* = .001) was 10.83. The degrees of freedom of the multistate single-trait and bi-factor models were equivalent. When comparing model fits between multistate single-trait and bi-factor models, care should be paid; fits of bi-factor models are generally superior to those of higher-order factor models, including multistate single-trait models, as bi-factor model provide less constrained solutions^35^.

We adopted robust maximum likelihood estimation (MLR), which provides scaled chi-square test statistics robust to non-normality and robust standard errors. Factor loadings of RSFC in the measurement models represent how strongly RSFC in a scan is associated with the common latent factor of RSFC; higher factor loadings can represent more reliable measurement of RSFC.

### Measurement models of RSFC

#### Comparisons between the single-trait model and multistate single-trait models with and without method factors

**Fig. 2:**
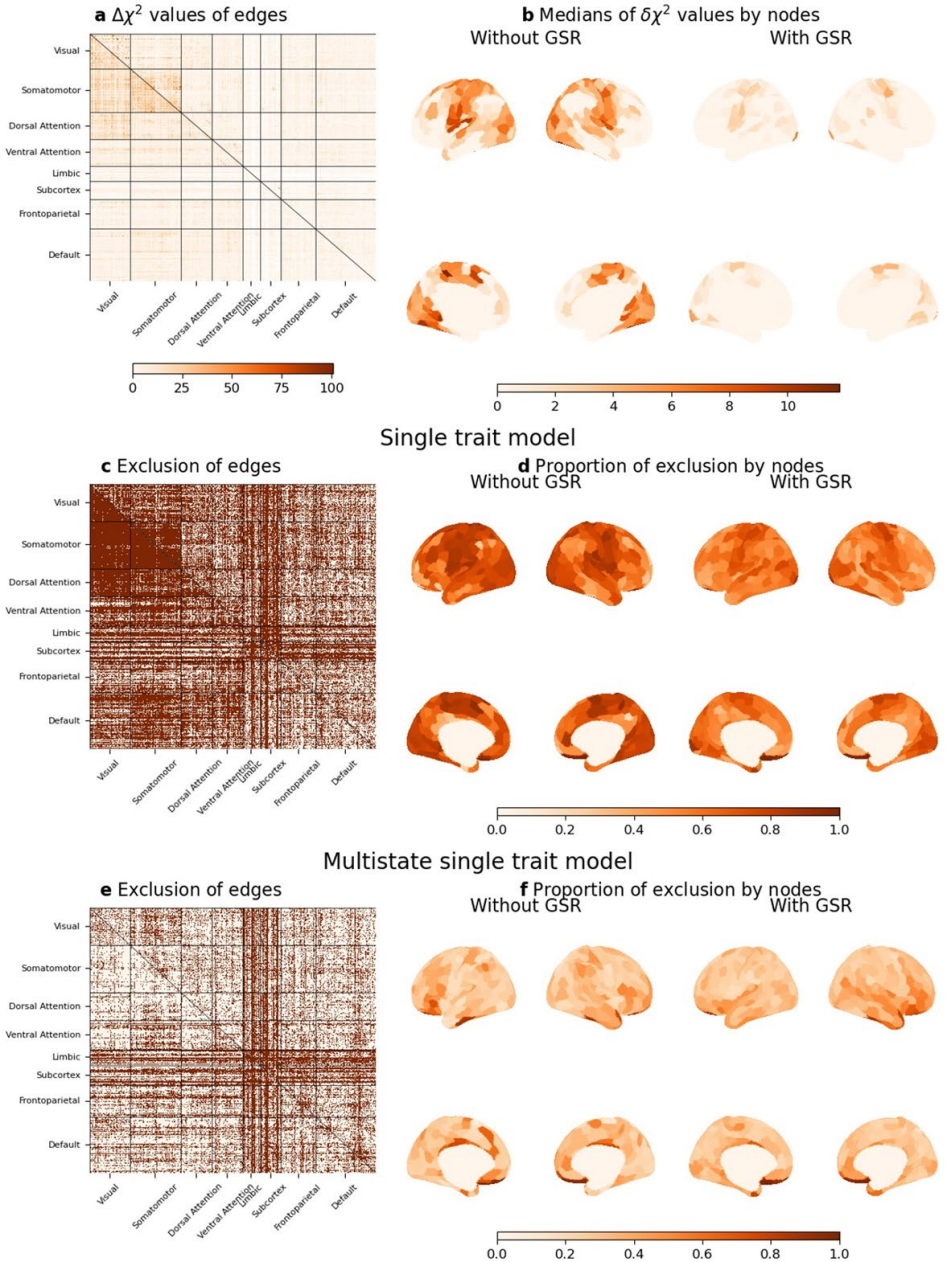
Comparisons between measurement models of RSFC without covariates in dataset 1 (*N* = 203). (**a**,**b**) Comparisons between single-trait and multistate single-trait models. (**a**) Heatmap of chi-square difference (*Δχ*^2^) values of edges. (**b**) Heatmaps of medians of chi-square difference values of edges by nodes. (**c**,**d**) Results of single-trait model. (**e**,**f**) Results of multistate single-trait model. (**c**,**e**) Heatmaps of removed edges, where brown represents removed edge. (**d**,**f**) Heatmaps of proportions of removed edges per nodes. Heatmaps above diagonals represent results of GSR condition and those below diagonal results of no-GSR condition. The criteria of model selection were not applied (see ‘Model evaluation’ in Method).

First, we compared the model fit (chi-square values) of the single-trait model without state effects (Fig. 1a) to the multistate single-trait model (Fig. 1b) using a dataset 1 (*N* = 203), across 93,096 RSFC edges, representing combinations of 400 cortical and 32 subcortical regions of interest (ROIs)^36,37^. Improvements in model fit were generally more pronounced for edges within sensorimotor (visual and somatomotor) networks than for higher-order control networks (dorsal attention, ventral attention, frontoparietal, and default), particularly when global signal regression (GSR) was not applied (Fig. 2a and b). These results suggest the importance of modeling measurement errors for accurate interpretation of RSFC. Additional fit indices, including the standardized root mean square residual (SRMR), root mean square error of approximation (RMSEA), and comparative fit indices (CFA), are presented in Supplementary Fig. S1, with cutoff values adopted in the present study^38^. Fits in certain number of edges did not reach the cutoff values, as described in Fig. 2c; model fits were worse in RSFC related to limbic network and subcortex.

We identified edges where measurement models failed to be established in the single-trait (Fig. 2c and d) and multistate single-trait models (Fig. 2e and f) (see ‘Model evaluation’ in Methods). In the single-trait model without state effects, measurement models failed to be established for many edges, particularly those associated with sensorimotor networks. In contrast, measurement models were established for many edges when using the multistate single-trait models. These findings suggest that a state factor is essential for establishing latent RSFC across two consecutive days, especially for sensorimotor networks.

To investigate method effects, we built multistate single-trait models incorporating method factors related to the order of the measurement days (Fig. 1c), using samples with repeated measurements with a counterbalanced order of phase encoding directions (*N* = 199 in each dataset). We found that many edges showed near-zero standardized factor loadings, although other edges showed negative or positive loadings (Supplementary Fig. S2); of note, the method factor is not required for many edges. Finally, we adopted multistate single trait model without method effects in the following analyses.

#### Comparisons between multistate single-trait and bi-factor models

Bi-factor models, in which a first-order latent common factor and orthogonal state (day) factors are assumed, could be considered as a possible alternative measurement model^31^ (Fig. 1d). While day factors are assumed to be independent of general factors in bi-factor models, common latent factors underlie day factors in the multistate single-trait models. Both models are theoretically plausible; therefore, we empirically compared the models in terms of standardized factor loadings and model fits.

Our analysis results showed that many edges showed near-zero loadings on the group (day) factor in a bi-factor model (Supplementary Fig. S3d). Near-zero standardized factor loadings in bi-factor models were particularly apparent in RSFC related to limbic network and subcortex (Supplementary Fig. S4). The fits of both models were very highly correlated, while the fits of bi-factor models were superior to those of multistate single-trait models in general, before removing edges which did not pass the model selection criteria (Supplementary Fig. S5a), which is consistent with previous findings^35^. However, after removing edges not passing the criteria, many of which were related to limbic network and subcortex (Fig. 2e), the model fits were almost equivalent (Supplementary Fig. S5b). These results suggest that both models were equally effective at measuring RSFCs after appropriate model selection, but could not appropriately measure the day effects in some RSFCs related to the limbic network and subcortex.

#### Variance decomposition of RSFC

**Fig. 3:**
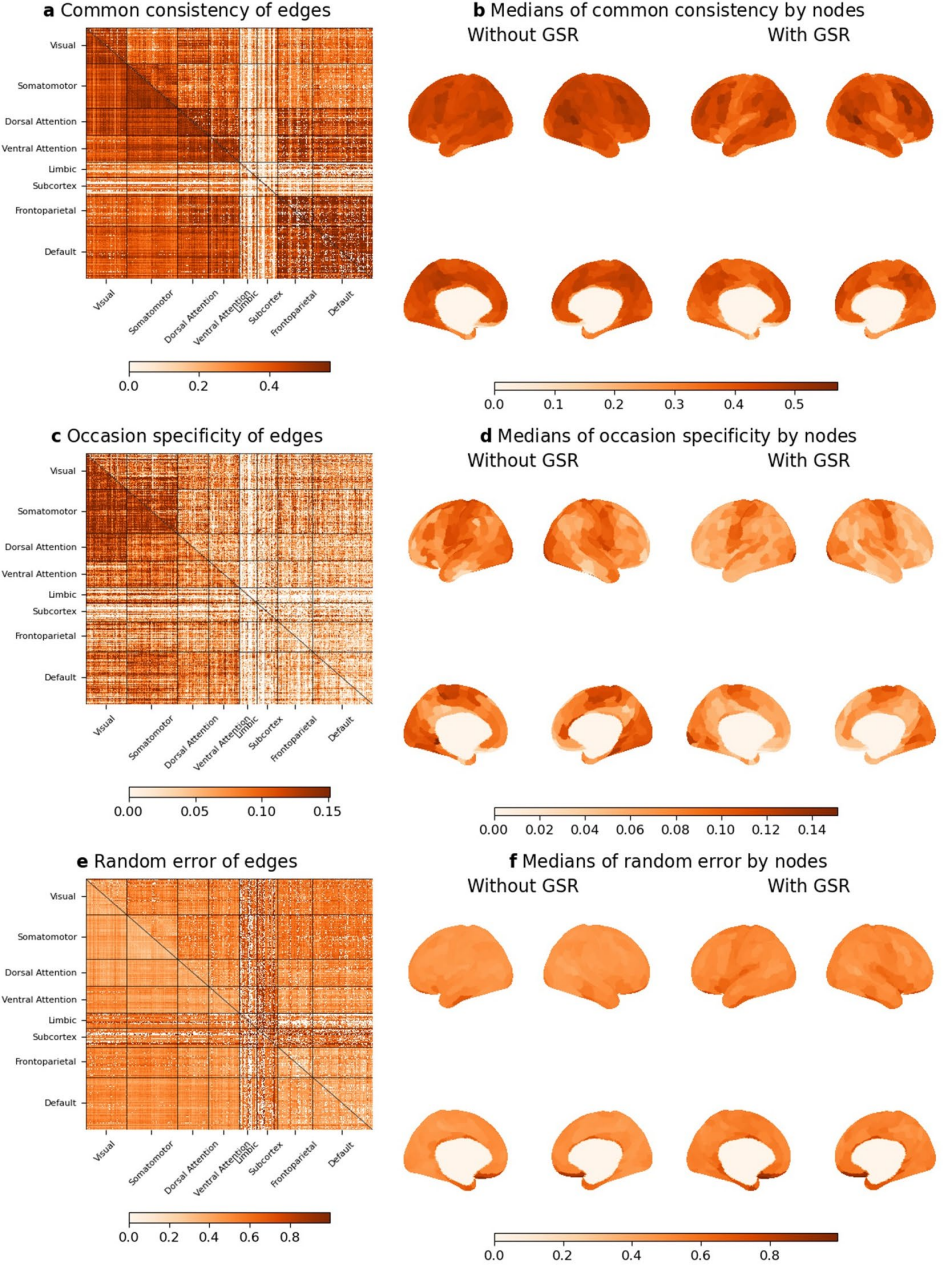
Variance decompositions of RSFC in dataset 1 (*N* = 203). Common consistency, occasional specificity, and random error of edges (**a**,**c**,**e**) and their medians by nodes (**b**,**d**,**f**). Common consistency, occasional specificity, and random error of edges were calculated by averaging values of four indicators in the measurement models. Model selection was not applied (see ‘Model evaluation’ in Method). Heatmaps above diagonals represent results of GSR condition and those below diagonal results of no-GSR condition.

We investigated the trait, state, and error components of RSFC using multistate single-trait models in each indicator. We calculated the common consistency (representing trait effects), occasional specificity (representing state effects), and random error of RSFC, as estimated from the multistate single-trait models. First, we evaluated the differences in trait, state, and error effects between scans. Importantly, we found that error effects significantly increased, while trait and state effects were significantly reduced in the second scan in each day, as shown in Supplementary Fig. S6. Although the absolute values of these effects were significantly different between scans, they were highly correlated (*r* > .77) in all conditions. Subsequently, we calculated the mean values of trait, state, and error effects, as illustrated in Fig. 3a–f, respectively. These results were very similar with those obtained from subjects with counterbalanced measurements (Supplementary Fig. S7). We found that common consistency was generally higher than occasional specificity, as clearly shown in Supplementary Fig S6. Typically, common consistency was greater for RSFC within networks compared to between networks (mean difference = 0.11). RSFC between sensorimotor networks and higher-order cognitive networks (dorsal attention, ventral attention, frontoparietal, and default) exhibited lower common consistency. The patterns observed for occasional specificity were similar to those of the chi-square difference tests, with occasional specificity tending to be higher in sensorimotor networks. Random errors were lower for RSFC within networks than between networks, which aligns with the observed tendencies in common consistency. Additionally, random errors of RSFC between sensorimotor and cognitive networks were higher, which similarly aligns with the lower common consistency observed for these connections.

Overall, GSR resulted in lower trait (mean difference = -0.040) and state (mean difference = -0.017) effects but higher error effects (mean difference = 0.056). Specifically, GSR reduced trait effects in RSFC within sensorimotor networks (*t* = -61.3, mean difference = -0.051), between sensorimotor networks (*t* = -83.3, mean difference = -0.084), and between sensorimotor and cognitive networks (*t* = -165.3, mean difference = -0.064). Conversely, GSR slightly increased RSFC within (*t* = 31.7, mean difference = 0.021) and between (*t* = 4.1, mean difference = 0.002) cognitive networks. Similar results were obtained in the validation dataset for the aforementioned analyses (Supplementary Figs. S8 and S9).

### Measurement reliability of phenotype scores

We assessed the omega measurement reliability of the phenotype measures (Table 1) using CFA based on the congeneric unidimensional measurement model (Fig. 1e). We observed that some measures, including the Total and Fluid scales from the NIH Toolbox and the Externalizing scale from the Achenbach Adult Self-Report (ASR), exhibited relatively low reliability (below 0.70), which could impact subsequent analyses.

**Table 1:** Omega measurement reliabilities of phenotype scores.

a NIH Toolbox				
Total	Fluid	Crystal		
0.661 (0.693)	0.656 (0.625)	0.831 (0.787)		
b ASR				
All	Internalizing	Externalizing	Others	
0.873 (0.906)	0.751 (0.798)	0.689 (0.753)	0.758 (0.858)	
c NEO-FFI				
Neuroticism	Extraversion	Openness	Agreeableness	Conscientiousness
0.840 (0.868)	0.779 (0.805)	0.753 (0.770)	0.774 (0.765)	0.814 (0.827)

Omega measurement reliability represents ratio of true score variance, which is free from measurement error, to total score variance, which includes error variance. Values out of and within parenthesis represent omega measurement reliabilities obtained in datasets 1and 2, respectively.

### RSFC-phenotype associations

**Fig. 4:**
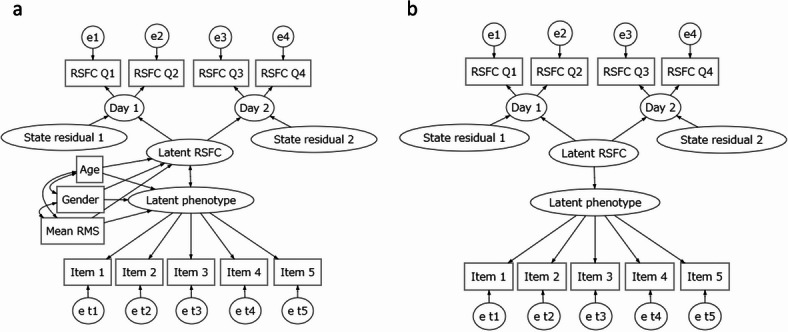
Examples of SEM used in the present study. (**a**) Example of SEM to estimate RSFC-phenotype association correcting measurement error attenuation. (**b**) Example of SEM to predict phenotype from RSFC through operative prediction using item or subscale scores as response variables. In each model, variances of latent variables were fixed to 1 and factor loadings of RSFC were equally constrained while those of phenotypes can vary. Covariates were not included in model (**b**) to avoid affecting model performance due to the different ways of treating covariates between competing models.

We used the full sample (*N* = 861) for investigating RSFC-phenotype associations, considering the large sample size requirements for modeling both RSFC and phenotypes. In these analyses, we accounted for state and error effects in RSFC, as well as test-, subscale-, or item-level measurement errors in psychological phenotypes (Fig. 4a).

#### Improvement factors in RSFC-phenotype associations

SEM revealed that RSFC-phenotype associations were, on average, 1.18–1.51 times stronger than those obtained using average scores of RSFC and phenotypes, and this improvement was nearly linear (Supplementary Fig. S10). Correlations estimated using SEM were strongly correlated with those obtained using average scores, which ranged from 0.88 to 0.99. The medians and means of improvement factors were 1.31 and 1.31 in no-GSR condition, and 1.33 and 1.33 in GSR condition, respectively. These results indicate that state and random effects in RSFC, along with test-, subscale-, or item-level measurement errors in psychological phenotypes, attenuated RSFC-phenotype associations by 15.3% (1–1/1.18) to 33.8% (1–1/1.51).

**Fig. 5:**
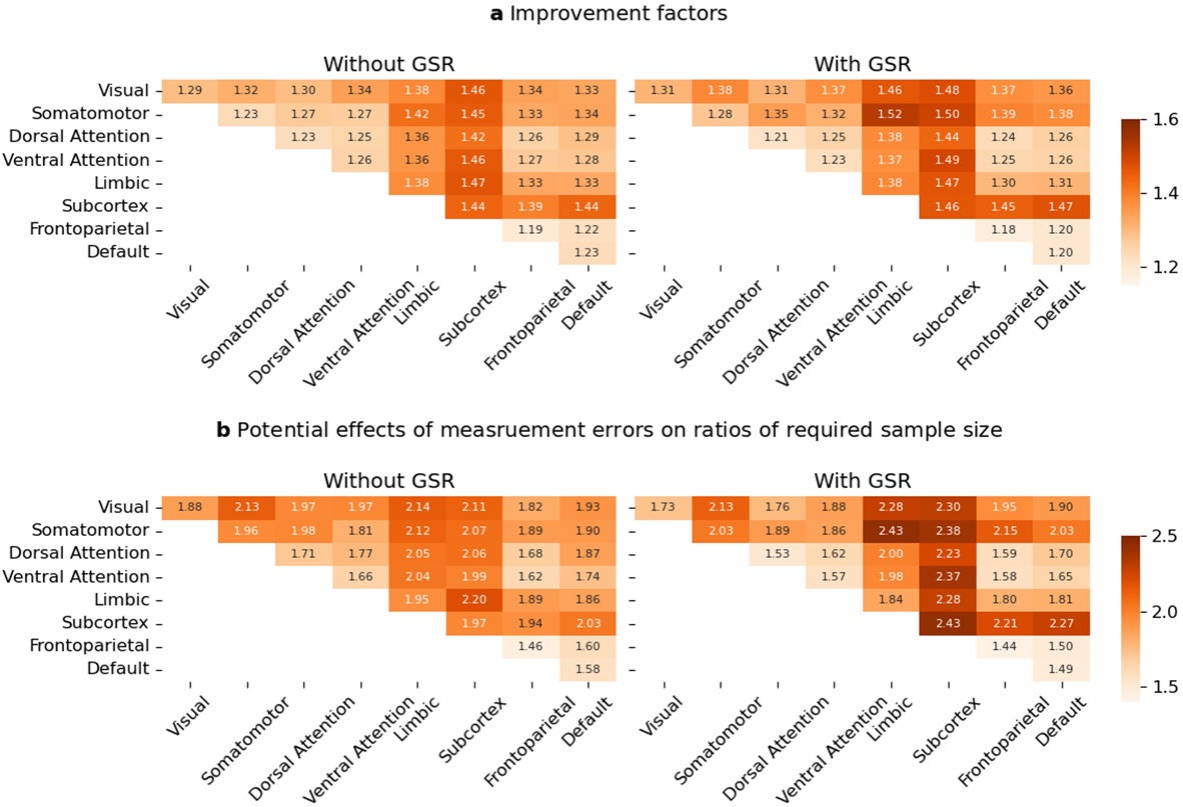
Results of RSFC-phenotype associations. (**a**) Means of improvement factors across the three types of phenotypes. Improvement factors represent linear regression slope of corrected association estimated in SEM on uncorrected association estimated in analyses on average scores of RSFC and phenotypes. (**b**) Potential effects of measurement errors on ratios of required sample sizes. The ratios were calculated by comparing the required sample size calculated from median RSFC-phenotype association estimated in SEM (corrected association) and those calculated from the associations estimated in analyses on aggregated scores (uncorrected association) across the phenotypes.

The means of improvement factors for combinations of networks across the 12 scales in the three types of the phenotypes and within each phenotype are presented in Fig. 5a and Supplementary Fig. S11, respectively. Improvement factors were higher in limbic networks and subcortical regions due to lower trait effects. We found that improvement factors were generally larger in RSFC involving sensorimotor networks than in those involving cognitive networks. Consistent with the variance decomposition results, while GSR tended to lead to slightly higher improvement factors for RSFC within sensorimotor networks (*β* = 0.03, 95% CI [-0.01, 0.06]), between sensorimotor networks (*β* = 0.07, 95% CI [0.03, 0.10]), and between sensorimotor and cognitive networks (*β* = 0.04, 95% CI [0.03, 0.06]), it led to lower improvement factors for RSFC within (*β* = -0.02, 95% CI [-0.04, -0.01]) and between (*β* = -0.02, 95% CI [-0.07, -0.03]) cognitive networks.

#### Effects of measurement errors on sample size requirements

We conducted post-hoc power analyses using correlations estimated in aggregated analyses and SEM to reveal the effects of measurement errors on required sample size to detect RSFC-phenotype associations. We calculated ratios of the required sample sizes and revealed that the effects of measurement errors differ between combinations of networks (Fig. 5b). Except for RSFC containing limbic network and subcortex, we found that in RSFC containing sensorimotor networks, the ratios of required sample size ranged from 1.81 to 2.13 and from 1.73 to 2.15 in no-GSR and GSR conditions, respectively. Meanwhile, in RSFC containing only cognitive networks, they ranged from 1.46 to 1.87 and from 1.44 to 1.70 in no-GSR and GSR conditions, respectively (Fig. 5b). The means of ratios of required sample sizes in combinations of networks were 1.90 and 1.93 in no-GSR and GSR conditions, respectively.

### RSFC-phenotype predictions

#### Regularized factor scores regressions

This section aimed to extend the findings in the “Measurement models of RSFC” (Fig. 1b) and “Measurement reliability of phenotype scores” (Fig. 1e) sections, where measurement models of RSFC and phenotypes were separately estimated using datasets 1 and 2. Although simultaneous estimation approaches including bias corrected factor score^39^ may be effective at ameliorating bias, their effects on variance in the machine learning framework remain unclear, and this topic seems beyond the purpose of the present study. We used a ten-repeated split-half cross-validation scheme with unrelated individuals (*N* = 407), extending the method used by McCormick et al.^19^using Pearson’s correlation (*r*) and coefficients of determination (*R*^2^) as performance metrics, with regularized ridge regression as the prediction model. First, we calculated validity coefficients, representing the correlation between latent factors and their factor score estimates in RSFC and phenotypes^25,40^. The validity coefficients for RSFC, excluding those related to limbic networks and subcortex^40 ^and for phenotypes were deemed sufficient for subsequent analyses using factor score estimates. These validity coefficients are reported in Supplementary Fig. S12 and Table S1.

**Fig. 6:**
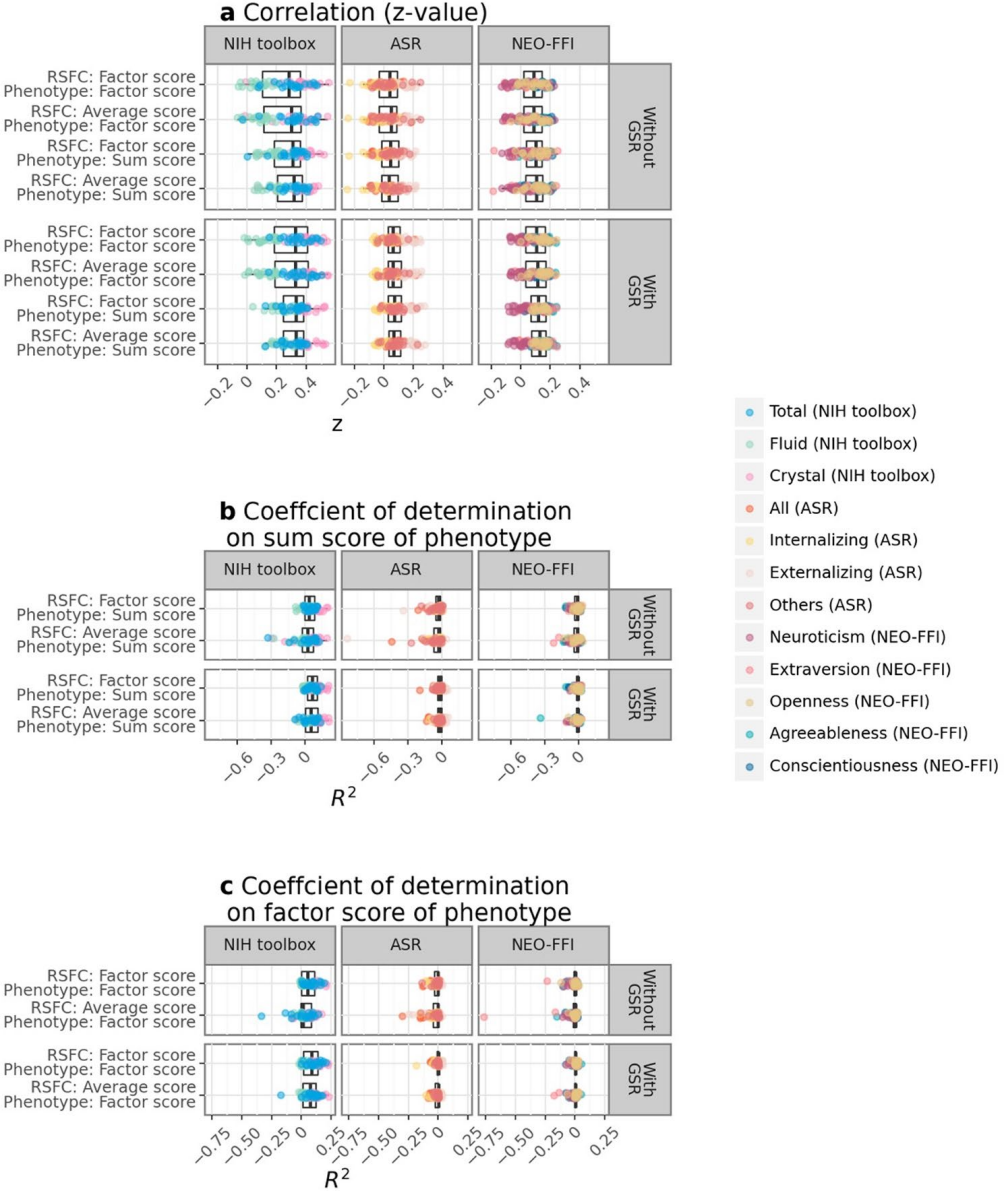
Comparisons of predictive performance using average scores or factor score estimates of RSFC as predictors and sum scores or factor score estimates of phenotypes as target variables. (**a**) Correlation (z-value) as a performance metric. (**b**,**c**) Coefficients of determinations (*R*^2^) using sums of squares as a performance metric. (**b**) Sum scores of phenotypes as target variable. (**c**) Factor score estimates of phenotypes as target variable. Since the sum scores and factor score estimates of target variables exhibit different variances, the results are independently shown in coefficients of determinations.

We observed that prediction accuracy was higher for cognitive measures than for mental health and personality measures as well as in the GSR than in no-GSR condition (Fig. 6). While the correlation coefficients did not significantly differ between predictive models using factor score estimates and average scores of RSFC, the *R*^2^ values were higher when using factor score estimates of RSFC as predictors compared to average scores, particularly for cognitive measures in the no-GSR condition (Fig. 6). Specifically, when predicting factor score estimates of phenotypes, the $\:\widehat{\varDelta\:}$*R*^2^ is 0.040 (0.012), 0.012 (0.004), and 0.011 (0.004) for the NIH Toolbox, ASR, and NEO-FFI, respectively. When predicting sum scores of phenotypes, the $\:\widehat{\varDelta\:}$*R*^2^ is 0.037 (0.014), 0.016 (0.002), and 0.007 (0.005) for the NIH Toolbox, ASR, and NEO-FFI, respectively. Values outside and inside the parentheses represent results for the no-GSR and GSR conditions, respectively. These results suggest that factor score estimates of RSFC resulted in more precise estimates of absolute values compared to using average scores of RSFC (Supplementary Fig. S13).

Conversely, we found that using factor score estimates of phenotypes as target variables resulted in lower predictive correlations, particularly for cognitive measures. Specifically, factor score estimates of RSFC yielded an $\:\widehat{\varDelta\:}z$ of -0.026 (-0.019), -0.003 (-0.009), and -0.011 (-0.011), while average scores of RSFC yielded an $\:\widehat{\varDelta\:}z$ of − 0.035 (-0.021), − 0.004 (-0.007), and − 0.011 (-0.012) for the NIH Toolbox, ASR, and NEO-FFI, respectively. Values outside and inside parentheses represent results in the no-GSR and GSR conditions, respectively.

#### Combination of operative prediction and regularized ridge regressions

**Fig. 7:**
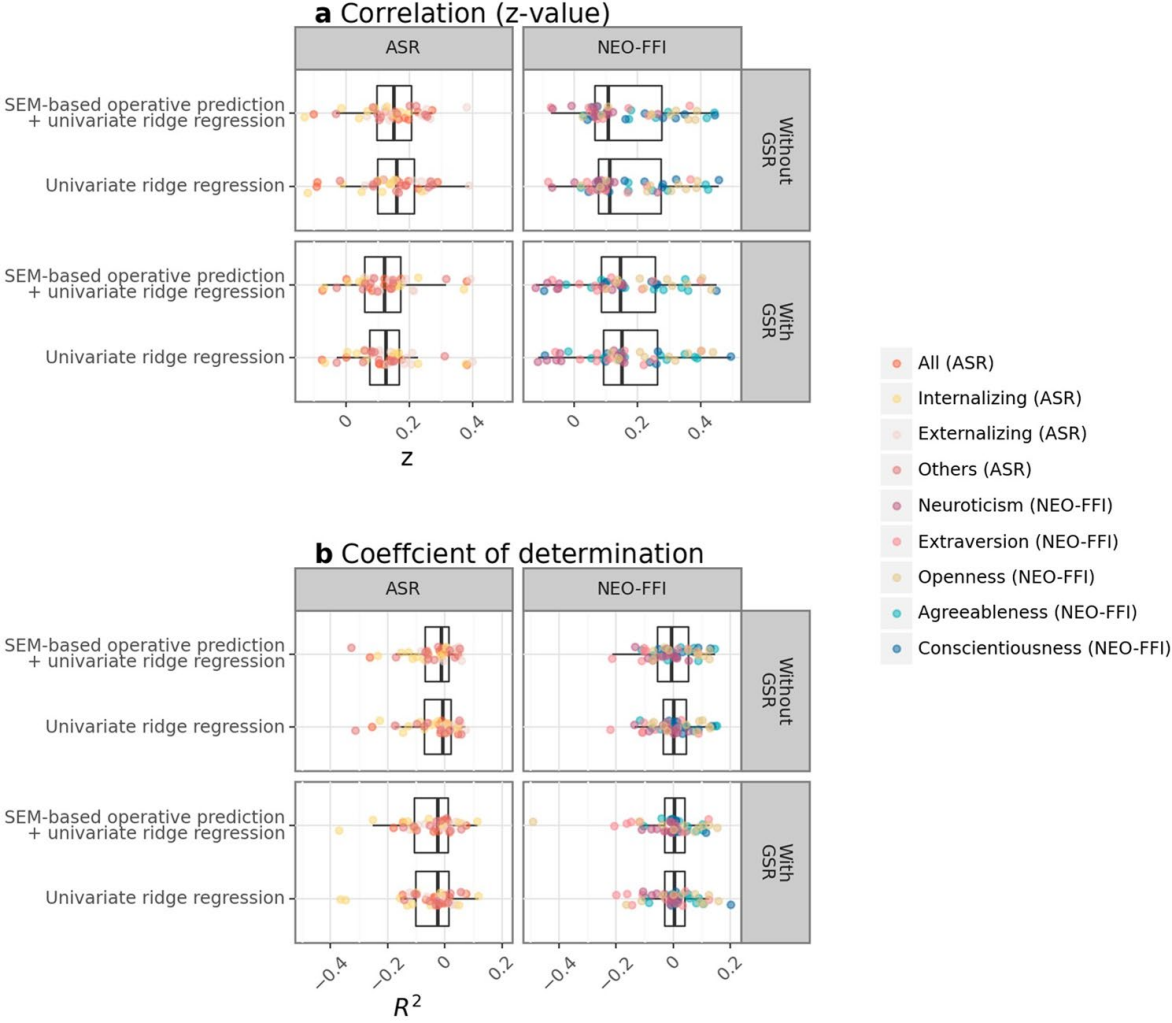
Comparisons of predictive performance of combinations of SEM-based operative prediction and univariate ridge regressions and simple univariate ridge regressions. (**a**) Correlation (z-value) as a performance metric. (**b**) Coefficients of determinations (*R*^2^) using sums of squares as a performance metric.

This section aimed to extend the results of the “RSFC-phenotype associations” section, in which simultaneous estimation of RSFC and phenotype was conducted using the full sample (*N* = 861). To examine the predictive performance of operative prediction, we used a 10-fold cross-validation scheme considering the family structure. We used CB-SEM in which the latent factor of RSFC predicted the latent factor of the phenotype (Fig. 4b). After obtaining predicted scale scores by summing the predicted item or subscale scores, we trained a univariate ridge regression model with the predicted scale score as the predictor and the true scale score as the response variable. These operations were conducted to obtain a single predictive value from 93,096 predictive values of phenotypes scores obtained through operative prediction. We compared the predictive accuracy of (i) predictive models that combined operative prediction with univariate ridge regressions described above and (ii) simple univariate ridge regressions where scale scores were directly predicted. The combined approach of operative prediction and univariate ridge regression did not outperform simple univariate ridge regression (|$\:\widehat{\varDelta\:}z$| < 0.01, |$\:\widehat{\varDelta\:}$*R*^2^| < 0.01) (Fig. 7).

## Discussion

This study investigated the state, trait, and error effects on RSFC, considering the influence of GSR, using the latent state-trait models. Variance decomposition of RSFC through repeated measurements is required in investigation of RSFC-phenotype relationships, as the within-individual variability of RSFC may inhibit the identification of stable relationships^7^. Moreover, we examined the RSFC-phenotype associations through SEM, correcting for state and error effects in RSFC, as well as test- (NIH toolbox), subscale- (ASR), or item-level (NEO-FFI) measurement errors in phenotype scores. Finally, we investigated the predictive accuracy using CFA or SEM.

Consistent with previous studies^5,13–15 ^our findings indicate that (i) state effects on RSFC are lower than trait effects; (ii) trait effects are lower in sensorimotor than in cognitive networks; and (iii) state effects are higher in sensorimotor than in cognitive networks. Our results also demonstrated that applying a multistate single-trait model to RSFC can provide valid estimates of trait, state, and error effects. In this study, GSR reduces trait effects in RSFC related to sensorimotor networks, which aligns with the findings of a previous study^41^. Importantly, corrected associations estimated using SEM revealed that RSFC-phenotype associations are attenuated by measurement errors in RSFC, including state effects, as well as measurement errors in phenotypes. Specifically, these attenuations were more pronounced in RSFC related to sensorimotor networks. Improvement factors, which compared RSFC-phenotype associations with and without corrections for measurement error, ranged from 1.19 to 1.51, and were higher in sensorimotor networks, especially under GSR. These improvement factors suggest that measurement errors in RSFC and phenotypes can attenuate associations by 15–34%. Post-hoc power analysis indicated that these attenuations could lead to a requirement of approximately double the sample size for detecting RSFC-phenotype associations. In this study, the degree of attenuation in RSFC-phenotype associations due to measurement error were higher than those reported by Teeuw et al.^18 ^who applied measurement models to only two indicators of RSFC (RSFC in day 1 and 2) and reported a range from 1.09 to 1.35 (mean 1.19). This discrepancy highlights the importance of considering a wide range of error effects, including those of state effects of RSFC and test-, subscale-, or item-level measurement errors of phenotypes. Finally, although we used scores of RSFC and phenotypes from a single time point (RSFC measured over two consecutive days, and phenotypes on a single day), controlling transient errors across time by incorporating repeated measures could further enhance estimates of RSFC-phenotype associations^12,42^.

The variance decomposition adopted in the present study was similar to the intra-class effect decomposition (ICED), in which variances of latent constructs estimated from brain measures are decomposed into several effects, including session and day effects, through a bi-factor measurement model^31^. Both the latent state-trait model, a form of higher-order factor model, adopted in the present study and bi-factor measurement models could be used in variance decompositions, with different conceptualizations of RSFC. We found that the model fits were almost equivalent between the models after selecting appropriate models. As described in the ‘Comparisons between multistate single-trait and bi-factor models’ section, models should be chosen based on the theoretical conceptualization of RSFC. Interestingly, we found that while trait and state effects were reduced, error effects increased in the second scan in each day through multistate single-trait models. Variance decomposition of indicators in the present study provided practical insights: more stable estimates of RSFC could be obtained in the first run in a day than in the second. This finding also has implications for ICED; instead of a parallel model, where equal error variances and factor loadings are assumed, more flexible models, including tau-equivalent and congeneric models, where different error variances and/or factor loadings are assumed, could be used to measure RSFC with repeated measurements in a day when empirical unequal variances are found. Future studies should investigate the causes of increasing error effects in a day, including individual factors such as sleepiness and fatigue as well as scanner-related factors, and controlling them may lead to more stable estimates of RSFC and clinical biomarkers.

The findings of the present study may also contribute to power calculation and sample size planning for future studies aiming to detect RSFC-phenotype associations; measurement error leads to requirement of about double sample size in general (Fig. 5). Previous studies have also shown that the RSFC related to somatomotor networks could be candidate stable biomarkers of mental disorders^43,44^, and especially careful attention should be paid to detect the associations between clinical phenotypes and these RSFCs.

Previous studies have explored ways to improve predictive accuracy using CFA or SEM^19,21,23^. In this study, we found that factor score estimates of RSFC could lead to higher coefficients of determination using sums of squares (*R*^2^), particularly when predicting cognition without GSR, although the correlation coefficients did not differ significantly. This may be attributed to stronger measurement models (higher common consistency) under the no-GSR condition^26^. While a previous study has shown that latent FC in various task improves predictive power^19^, latent RSFC did not exhibit such improvement. This suggests that incorporating task-related FC may be necessary to achieve better predictive correlations. Furthermore, using factor score estimates of phenotypes as target variables resulted in lower predictive correlations, particularly for cognitive measures, possibly due to the lower quality of measurement in cognitive tasks (Table 1). Adopting factor score regressions or structural after measurements approach^45^ is a possible future directions to improve predictive performance, although more research is required to apply these methods in predictive modeling.

Although CB-SEM based operative predictions combined with univariate ridge regressions did not outperform simple univariate ridge regressions, further exploration of different modeling strategies, including regularized SEM with multiple RSFC as predictors^46^ or partial least squares SEM, with latent factors as linear composites^28 ^may improve predictive accuracy.

There are several limitations in the present study. First, we acknowledge the adoption of a unidimensional assumption in phenotype measures. Several phenotype measures, including the Total and Fluid scales from the NIH Toolbox and the Externalizing scale in the ASR, showed relatively low composite reliability estimates (< 0.70). The application of bifactor modeling, where group factors, as well as the common latent factors, are assumed to explain the heterogeneity of indicators^47 ^may be beneficial for more precise estimates of RSFC-phenotype associations^17^ and better performance in RSFC-phenotype predictions. Moreover, the modeling of the ordinal nature of item scores, although computationally intensive^48 ^should be considered in future research.

Second, it is plausible that measurement errors differentially affect performance in RSFC-phenotype predictions, since our study found that measurement errors differentially affect RSFC-phenotype associations between combinations of networks. Further research is needed to investigate potential differential predictive performance of RSFC across networks.

Third, although we focused on single-trait models in the present study, future studies may extend to multi-trait multi-method models, in which multiple latent trait and method factors explain observed RSFC. For example, one prior study on structural MRI revealed that multi-trait multi-method modelling on four regions of interest with three measurement methods lead to 12.5% explained variance of latent episodic memory in a large sample including older adults^49^. It may be possible that RSFCs could be explained by multi-trait factors, including networks and hemispheres. Furthermore, method factors, including phase encoding directions, may be included in some RSFCs.

Finally, the present study used RSFC data obtained across four scans; however, SEM can be applied to data from a single scan^18^. Future studies should investigate within-scan variability of RSFC by splitting one fMRI scan into multiple parts.

In conclusion, this study demonstrated that associations between stable trait factors of RSFC and psychological phenotypes, including cognition, mental health, and personality, are significantly attenuated by state and error effects in RSFC, as well as test-, subscale-, or item-level measurement errors in psychological phenotypes. The degrees of these attenuations were higher in sensorimotor networks compared to higher-order control networks. These attenuations can lead to a requirement of approximately double the sample size to detect RSFC-phenotype associations. We also found that regularized regressions using factor scores of RSFC as predictors led to higher coefficients of determination than when using average scores, while SEM-based operative prediction did not show similar improvements. Our findings support the idea that CB-SEM can be useful in correcting the attenuation of RSFC-phenotype associations, considering both state and error effects. Future research should explore more effective methods for RSFC-phenotype predictions utilizing SEM while addressing the impact of within-individual variability and measurement error. The present study will promote neuroscientific research using repeated measurements and SEM to identify more accurate RSFC-phenotype relationships, free from within-individual variability.

## Methods

### The Human Connectome Project

We used the publicly available dataset of resting-state fMRI after the ICA-FIX (FMRIB’s ICA-based Xnoiseifier) denoising^50,51^ of the Human Connectome Project Young Adult 1200 Subject release^20^. The dataset includes both fMRI and behavioral assessment data measured over two days from healthy young adults. Resting-state fMRI was acquired for 15 min per day using different phase encoding directions with counterbalanced order (right-left and left-right in day 1 and left-right and right-left in day 2 in many, but not all, subjects), resulting in a total scan duration of one hour. The details of preprocessing are shown in previous literatures on the HCP^52,53^.

#### Participants selection

We excluded participants whose at least one session included > 10% of volumes where the relative root-mean-squared displacement is > 0.25 mm to ameliorate the effects of motion artifact. We included participants with all phenotypic data described in ‘Phenotype measure’ and 861 participants (398 women [27.7 ± 3.6 years old] and 463 men [29.4 ± 3.6 years old]) were included.

We randomly split family to two datasets, and randomly subset one member from each family. Datasets 1 and 2 include 203 (108 women [30.4 ± 3.3 years old] and 95 men [27.6 ± 3.5 years old]) and 204 (106 women [29.6 ± 3.9 years old] and 98 women [27.3 ± 3.4 years old]) individuals, respectively. We examined whether the findings in dataset 1 were supported in dataset 2, with exploratory and validation analyses conducted in datasets 1 and 2, respectively. Since phase encoding directions and scan orders were not counterbalanced in some subjects, we created a subset of subjects with counterbalanced measurements in analyses focusing on measurement order.

#### Phenotype measures

We used three measures, namely the National Institute of Health (NIH) Toolbox measuring cognition^54^, the Achenbach Adult Self Report (ASR) measuring psychiatric and life function^55^, and the NEO Five-Factor Inventory (NEO-FFI) measuring personality^56^.

#### NIH Toolbox

The NIH Toolbox provides three summary scores: Total Cognition Composite (Total), Fluid Composite (Fluid), and Crystallized Composite (Crystal)^57^. Fluid Composite score is a normalized average score of five tests, including (i) Dimensional Change Card Sort, (ii) Flanker Inhibitory Control and Attention, (iii) Picture Sequence Memory, (iv) List Sorting Working Memory, and (v) Pattern Comparison Tests. Crystallized Composite is a normalized average score of two tests, including (vi) Picture Vocabulary and (vii) Oral Reading Recognition Tests. Total composite score is a normalized average score of the seven tests described above. Higher scores represent better cognitive functioning. We used unadjusted scores provided in the HCP. Since all the test scores were normalized in the HCP, we could not calculate composite scores from the test scores (see ‘Evaluation of performance of SEM-based operative prediction’).

#### Achenbach Adult Self Report (ASR)

The ASR provides ten subscale scores in the HCP, including (I) Anxious/Depressed, (II) Withdrawn, (III) Somatic Complaints, (IV) Thought Problems, (V) Attention Problems, (VI) Aggressive Behavior, (VII) Rule Breaking Behavior, (VIII) Intrusive, (IX) Other Problems, and (X) Critical Items. Internalizing and Externalizing scores are calculated by summing the scores of the subscales (I), (II), and (III) and those of the subscales (VI), (VII), and (VIII), respectively^55^. In addition to these scores, Thought, Attention, and Other Problems are calculated by summing the scores of the subscales (IV), (V), and (IX) in the HCP. Total score was calculated by summing all of the scales, excluding the Critical Items^55^. We used Total, Internalizing, and Externalizing, as well as Thought, Attention, and Other Problems scores, in accordance with the dataset in the HCP.

#### NEO Five-Factor Inventory (NEO-FFI)

The NEO-FFI provides five subscales, including Neuroticism, Extraversion, Openness, Agreeableness, and Conscientiousness^56^. Each subscale comprises of twelve items and subscale scores were calculated by summing the item scores. Participants answered the items using the 5-point Likert scale: 1 = Strongly Disagree, 2 = Disagree, 3 = Neither Agree or Disagree, 4 = Agree, 5 = Strongly Agree.

### Preprocesses of fMRI data

First, we excluded the first four volumes of each scan. Then, we applied spike regression to fMRI data with thresholds of relative root-mean-squared displacement being ≥ 0.25 mm to remove motion artifact. In the GSR condition, we calculated mean gray-matter signal using all grayordinates. It has been shown that mean-gray matter signal is very highly correlated with global signal^58 ^and we used mean gray-matter signal instead of global signal as a regressor in the GSR condition. Subsequently, we parcellated voxels using 400 cortical parcels^36^ and 32 subcortical parcels^37^. We calculated functional connectivity using these 432 parcels (93,096 edges) in each scan. When applying SEM, we used Fisher’s z-transformed values of RSFC in each session. We also calculated means of Fisher’s z-transformed values of RSFC in the four scans to compare RSFC-phenotype associations estimated from average scores of RSFC and phenotypes and those estimated in SEM.

### Measurement models of RSFC

We further compared several types of models to investigate state effects: single-trait model, multistate single-trait model^30^, multistate single-trait model with method factors of measurement order in days, and bi-factor model^31^ (Fig. 1a–d). In single-trait model, it is assumed that single latent trait factor explains variation of indicators (RSFC). This can be a baseline model without any state and method effects. In the multistate single-trait model, we assumed two state factors representing measurement days, which are orthogonal, and a one trait factor explaining state factors. In the multistate single-trait model with method factor of measurement order in days, we additionally assumed two method factors representing the measurement order in days. In the bi-factor model, we assumed orthogonal two state factors and one trait factor, which explains four indicators of RSFC. Model fits were further compared using difference values of chi-square statistics (likelihood-ratio tests).

When estimating models, we standardized factor variances to 1. We equally constrained factor loadings in the same types of factors (trait, state, and method factors), according to previous neuroscientific study applying latent state trait models^59^. We used *lavaan* package in R when applying the SEM^33^. We used maximum likelihood estimation, which provides asymptotically distribution free chi-square test statistics^60^ (MLR in *lavaan*) in all the analyses. Moreover, we used *semdiag*, an open-source web application for drawing path diagrams^61^.

#### Model evaluation

When evaluating measurement models of RSFC, we examined global fit indices and standardized factor loadings. Since global fit indices may not inform appropriateness of parameter estimates^62^ we have independently examined them. We adopted standardized root mean square residual (SRMR), root mean square error of approximation (RMSEA), and comparative fit index (CFI) as global fit indices^63^. We adopted cutoff criteria of SRMR < 0.08, RMSEA < 0.08, and CFI > 0.90 according to previous simulation study of SEM^38^ and study where SEM was applied to investigate brain-phenotype associations^18^. We excluded models where the first order factor loading of RSFC was > 0.90 or < 0.40, and the second order factor loading of RSFC was > 1 or < 0.70 in multistate single-trait model (Supplementary Fig. S3a, S3b). This selection criteria successfully excluded improper structural correlation when investigating RSFC-phenotype associations using the full sample (Supplementary Figs. S10 and S14) and led to model fits equivalent to bi-factor models (Supplementary Fig. S5).

#### Decomposition of variance of RSFC

Since multistate single-trait model showed better model fits than single-trait model, we adopted the former. In a multistate single-trait model, several types of coefficients can be calculated^11^ and we reported three types of indices: common consistency, occasion specificity, and random error. Common consistency and occasion specificity represent the proportion of trait and state effects to modeled score variance of RSFC, respectively. Random error represents proportions of variance not explained by state, trait, and method effects. Random error equals one minus sum of common consistency and occasion specificity, if no method effects are included in a model. These values were calculated in each indicator (four runs of RSFC), and the average values were presented in the results after examining the values of each run in the subjects with counterbalanced measurements.

We investigated effects of GSR on these variance estimates in cerebral cortex, classifying into sensorimotor (visual and somatomotor) and cognitive networks (dorsal attention, ventral attention, frontoparietal, and default), using paired *t*-tests. We did not investigate effects of GSR in limbic network and subcortex since many measurement models were not established in these regions (Fig. 2).

### Measurement reliability of phenotype measures

We calculated omega composite reliability^64^ of phenotype measures in both datasets 1 and 2, using a congeneric unidimensional model (Fig. 1e). Although no clear cutoff criteria exist for omega composite reliability, values > 0.70 are often considered adequate^65^.

### RSFC-phenotype associations

We excluded SEM by applying criteria of parameter estimates described in ‘Model evaluation’. We also excluded SEM where factor loadings of phenotypic indicators were < 0 or > 1.

#### Improvement factor

We calculated improvement factor of RSFC-phenotype association according to Teeuw et al.^18^. We conducted linear regression analyses with correlation estimated in analyses on aggregated scores (means of the *z*-values of RSFC and indicators of phenotype measures) controlling covariates (age, sex, and mean relative root-mean-squared displacement across four scans) as explanatory variable and structural correlation estimated in SEM (Fig. 4a) as response variable. The slopes in these regressions were improvement factors. We also calculated improvement factors in combinations of seven networks^29^ and subcortex to investigate differential effects of measurement errors between networks.

#### Post-hoc power analysis

We conducted post-hoc power analysis to investigate how measurement error attenuation led to increased sample size to detect RSFC-phenotype associations. We initially computed estimates of required sample sizes to find the RSFC-phenotype associations using correlations estimated from average scores of both RSFC and phenotypes and structural correlation estimated in SEM. Subsequently, we calculated the ratio of required sample size from these estimates in both no-GSR and GSR conditions, considering brain networks.

### RSFC-phenotype predictions

As a baseline model, we adopted ridge regression regressor with average scores of RSFC being predictors and sum scores of phenotype measures being target variables. We compared prediction accuracy of the two types of prediction methods to that of baseline models: one is ridge regressions using factor scores estimates (regularized factor scores regression) of RSFC and/or phenotype measures and the other one is combinations of operative prediction using SEM and ridge regressions. When conducting ridge regressions, we determined optimal hyperparameter of regularization (*α*) with nested cross-validation, ranging from 10^3^ to 10^7^. We reported z-transformed Pearson’s correlation and coefficients of determination using sums of squares (*R*^2^) as performance metric. *R*^2^ was defined as follows.

1 $$\:1-\:\frac{{\sum\:}_{i=1}^{n}{({y}_{i}-\widehat{{y}_{i}})}^{2}}{{\sum\:}_{i=1}^{n}{({y}_{i}-\stackrel{-}{y})}^{2}}$$

where $\:\widehat{{y}_{i}}$ was the predicted value of the subject *i*, $\:{y}_{i}$ was the corresponding true value, and $\:\stackrel{-}{y}$ represented average of the true values. *R*^2^ ranges from -∞ to 1 and did not correspond with squares of Pearson’s correlation.

When examining differences between predictive methods, we applied linear mixed-effects models (LMM) with metric as a response variable and predictive methods as explanatory variable in the three types of the phenotypes in no-GSR and GSR conditions. The repeats of cross-validation or folds were nested within scales when applying LMM. In all the analyses using factor scores or operative prediction, we excluded edges related to limbic network and subcortex since measurement models were not established in many edges in these regions (Fig. 2e) as model misspecification can lead to biased results^21,26^. We did not include covariates across the analyses owing to the different ways of treating covariates (i.e., while covariates are related to observed variables in baseline models they are related to latent variables in SEM), which can affect predictive performance.

#### Regularized factor scores regressions

We repeated split-half cross validation ten times, extending the split-half cross-validation scheme adopted byMcCormick et al.^19 ^with unrelated individuals (*N* = 407) who were randomly selected from each family. According to McCormick et al.^19 ^we calculated factor scores of RSFC and phenotype measures in training and test datasets independently through the CFAs without covariates. Subsequently, we calculated the validity coefficients^25,40 ^which represents the correlation between factor and factor scores, with values > 0.80 recommended and > 0.90 preferable for use in subsequent analyses^25,40^. Validity coefficients supported exclusion of RSFC related to limbic network and subcortex (Fig. S12). We imputed zeros in scores of RSFC where measurement models were not established in either training or test sets (see a section of ‘Model evaluation’).

We build the following four types of prediction models in the twelve phenotype; (i) models with average scores of RSFC as explanatory variables and sum scores of phenotypes as target variables (baseline model), (ii) models with factor scores of RSFC as explanatory variables and sum scores of phenotypes as target variables, (iii) models with average scores of RSFC as explanatory variables and factor scores of phenotypes as target variables, and (iv) models with factor scores of RSFC as explanatory variables and factor scores of phenotypes as target variables. Factor scores of RSFC were calculated through regression methods^66^ while those of phenotypes were calculated through the Bartlett method^67^ to avoid bias^68^. The ridge regression was applied in each combination.

#### Evaluation of performance of SEM-based operative prediction

We conducted 10-fold cross-validations where participants belonging to the same family were included in train or test sets using full sample (*N* = 861), as more sample sizes were required when building SEM. We compared the two types of predictive models: (i) univariate ridge regression, where scale scores were directly predicted (baseline model), and (ii) combination of SEM-based operative predictions and univariate ridge regression. We reported the results of the ASR and NEO-FFI since only the normed scores of the NIH toolbox were open in the HCP, and we could not calculate the scale scores from the test scores in the NIH toolbox. In (ii), we obtained predictive values through operative prediction in each RSFC and use these values in training of subsequent univariate ridge regressions with true scale score being response variable. We can predict subscale or item scores ($\:\widehat{\varvec{y}}$) in the Eq. (2), using parameter estimates in SEM with mean structures in operative prediction^21^,

2 $$\:\widehat{\varvec{y}}=\:{\widehat{\varvec{\mu\:}}}_{\text{y}}+\:{\widehat{\sum\:}}_{xy}^{\top\:}{\widehat{\sum\:}}_{xx}^{-1}({\varvec{x}}_{0}-{\widehat{\varvec{\mu\:}}}_{x})$$

where $\:{\widehat{\varvec{\mu\:}}}_{\text{y}}$ represents model-implied mean vector of response variable, $\:{\widehat{\sum\:}}_{xy}^{}$ represents model implied covariances of predictor and response variables (phenotype), $\:{\widehat{\sum\:}}_{xx}^{}$ represents model implied covariance matrix of predictor variables (RSFC), $\:{\varvec{x}}_{0}$ is a new observation of predictor value, and $\:{\widehat{\varvec{\mu\:}}}_{x}$ represents model-implied mean vector of predictor variable. While the operative prediction is robust to violation of normality assumption, it is sensitive to misspecified model^21^. Models with improper parameter estimates (see ‘RSFC-phenotype associations’) were excluded in operative predictions.

## Supplementary Information

Below is the link to the electronic supplementary material.

Supplementary Material 1

## Data Availability

The dataset analyzed in the present study is publicly available in https://db.humanconnectome.org/ after registration.
