# Supplementary material for: Effects of measurement errors on relationships between resting-state functional connectivity and psychological phenotypes
Source: Sci Rep. 2025 Aug 19;15:30316. doi: 10.1038/s41598-025-13105-0 (PMC12365047; doi:10.1038/s41598-025-13105-0)
Supplement: Supplementary file 1 — Supplementary Material 1 [file 41598_2025_13105_MOESM1_ESM.docx]

**Effects of measurement errors on relationships between resting-state functional connectivity and psychological phenotypes**

Tomosumi Haitani^1^, Yuki Sakai^1, 2^, Saori C Tanaka^1, 3, *^

1. ATR Brain Information Communication Research Laboratory Group, 2-2-2 Hikaridai Seika-Cho, Soraku-Gun, Kyoto 619-0288, Japan

2. Department of Psychiatry, Graduate School of Medical Science, Kyoto Prefectural University of Medicine, 465 Kajii-Cho, Kawaramachi-Hirokoji, Kamigyo-Ku, Kyoto 602-8566, Japan

3. Division of Information Science, Graduate School of Science and Technology, Nara Institute of Science and Technology, 8916-5 Takayama-Cho, Ikoma, Nara 630-0192, Japan

**
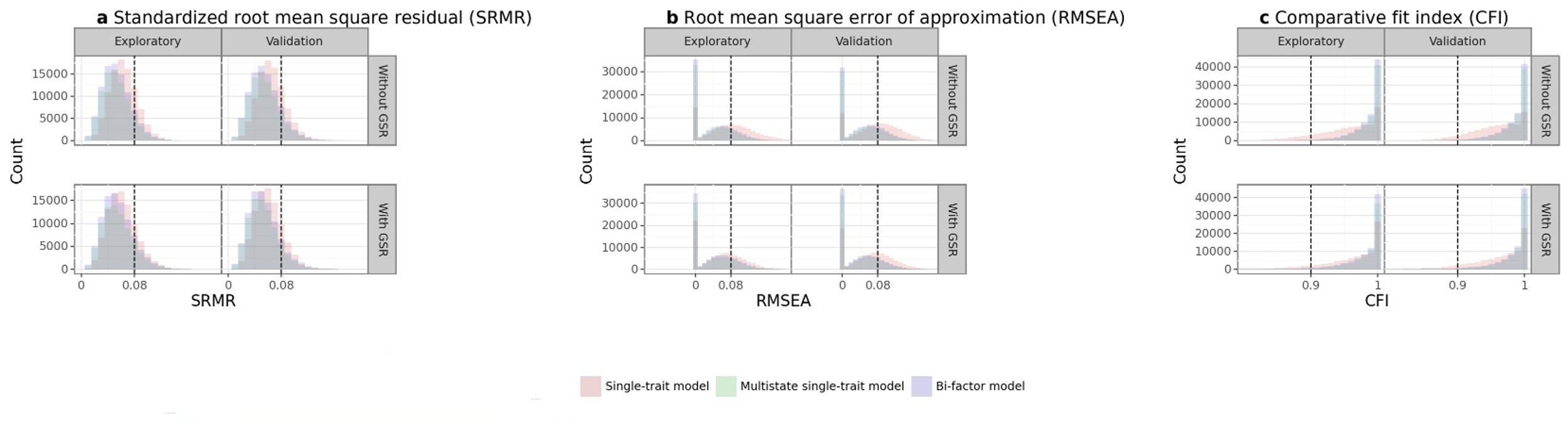
**

**Supplementary Figure S1:** Histograms of the fit indices of the single-trait, multistate single-trait, and bi-factor models. **a,** root mean square residual, **b,** root mean square error of approximation, **c,** comparative fit indices. Dashed lines represent the cutoff values adopted in the present study. Exploratory and Validation represent results for datasets 1 and 2, respectively. GSR represents global signal regression.

**
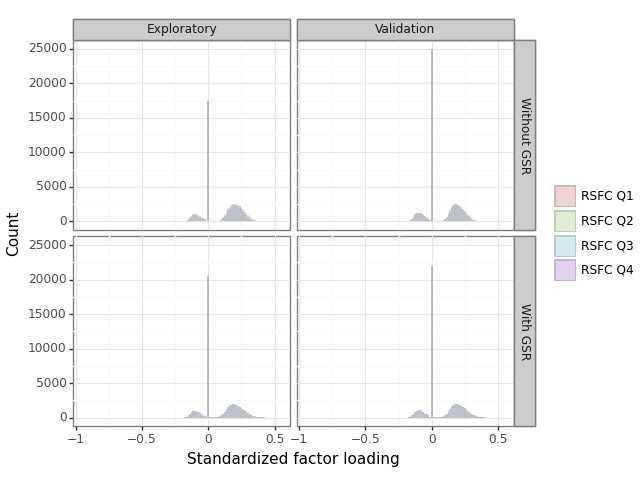
**

**Supplementary Figure S2:** Standardized factor loadings of the method factors representing the measurement order in days in the multistate single-trait model. Results for the individuals with repeated measurements of RSFC in counterbalanced order of phase encoding directions are shown (*N* = 199 in each dataset). Q1, Q2, Q3, and Q4 represent run 1 in day 1, run 2 in day 1, run 1 in day 2, and run 2 in day 2, respectively. Exploratory and Validation represent results for datasets 1 and 2, respectively. GSR represents global signal regression.

**
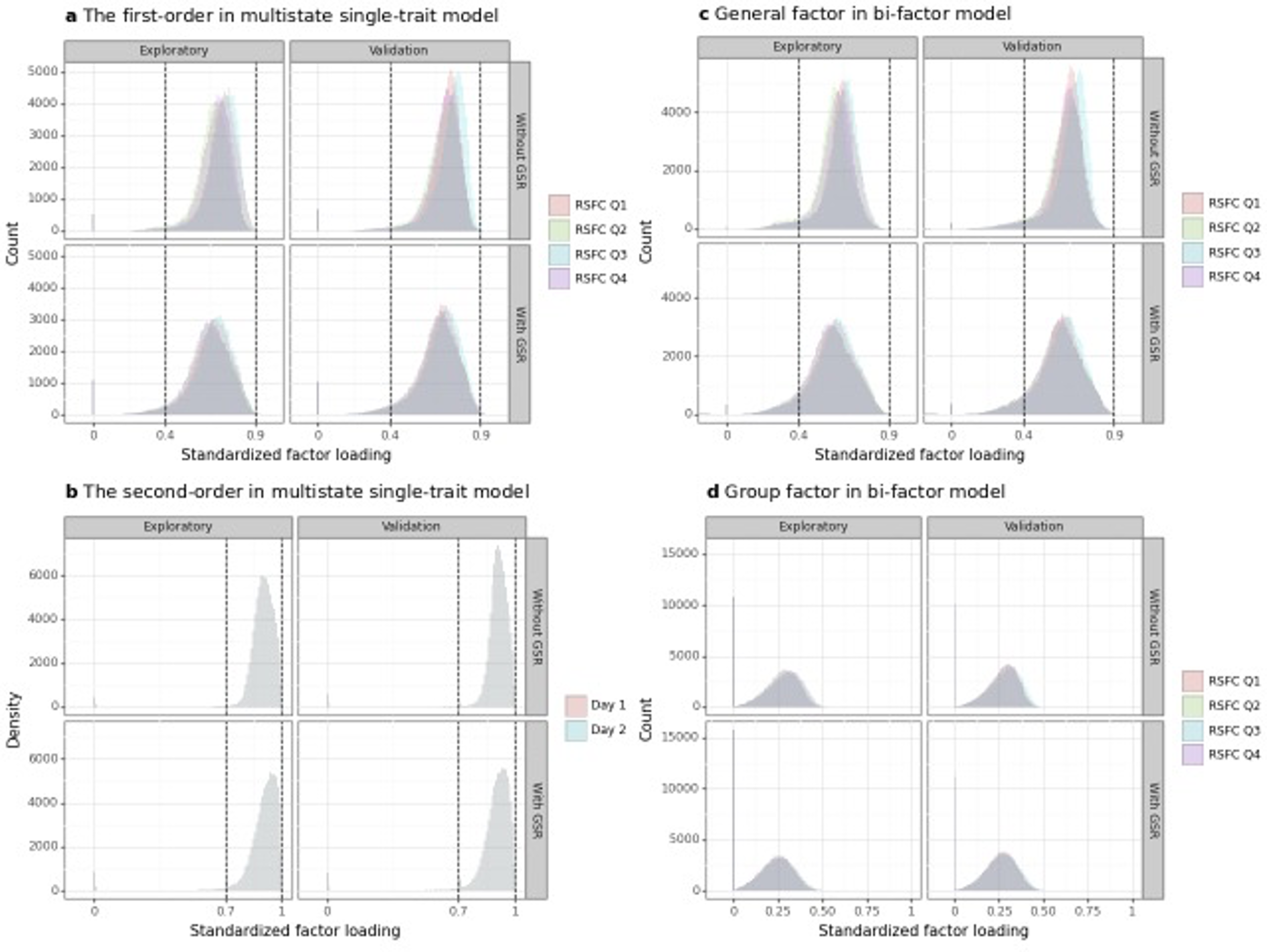
**

**Supplementary Figure S3:** Histograms of the standardized factor loadings in multistate single-trait (**a, b**) and bi-factor models (**c, d**). Q1, Q2, Q3, and Q4 represent run 1 in day 1, run 2 in day 1, run 1 in day 2, and run 2 in day 2, respectively. Exploratory and Validation represent results for datasets 1 and 2, respectively.


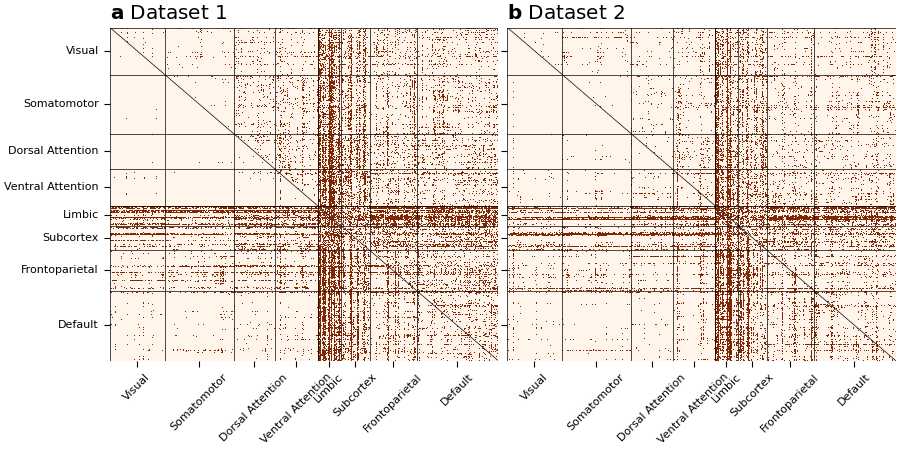


**Supplementary Figure S4:** Edges where the standardized factor loadings of group factors were near-zero in the bi-factor measurement model. Brown represents edges whose standardized factor loadings of group factors were near-zero.


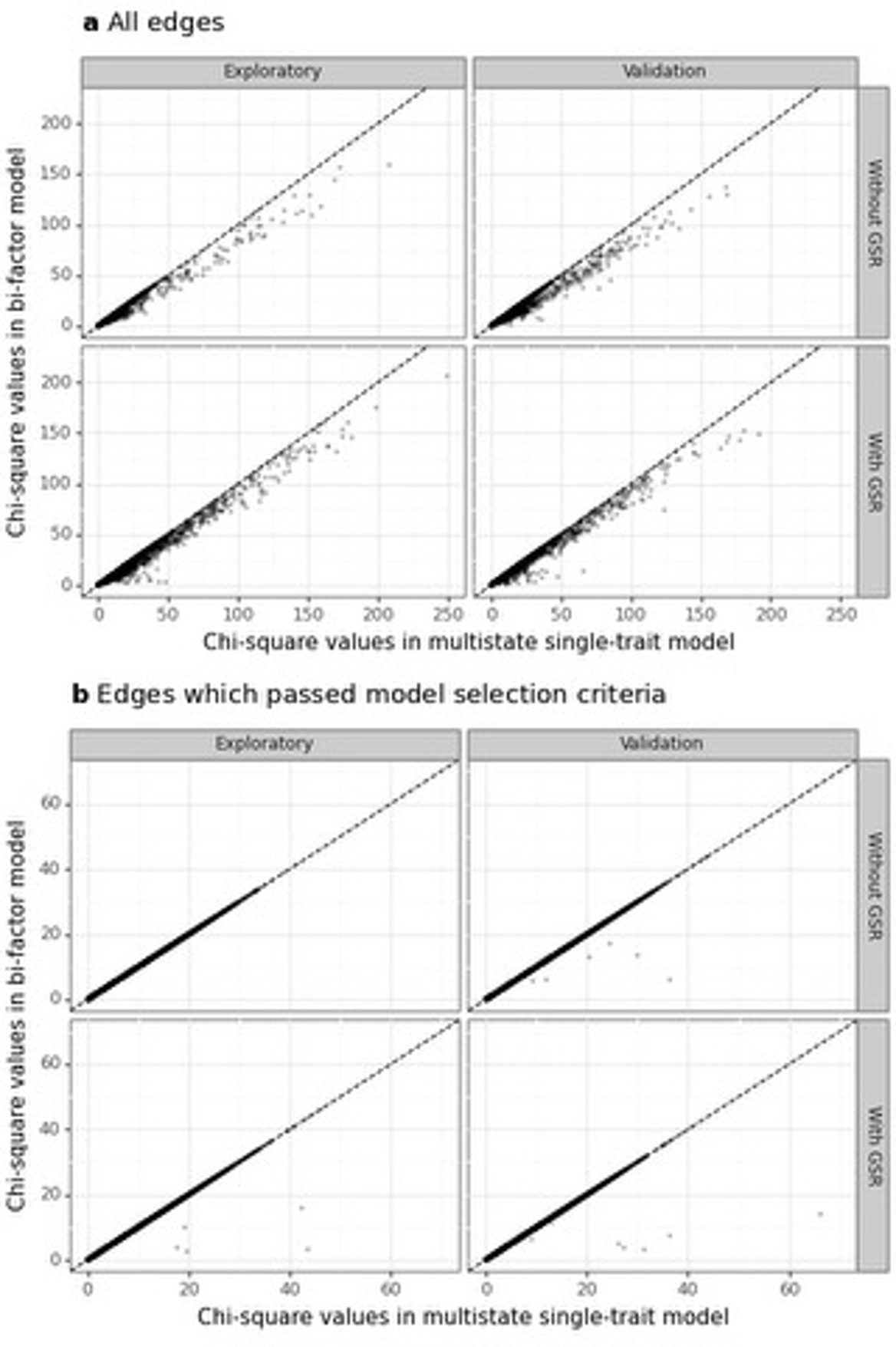


**Supplementary Figure S5:** Comparisons of model fits between multistate single-trait and bi-factor models. **a,** All edges. **b,** Edges which passed model selection criteria, described in ‘Model evaluation’ in Method in the main text. Exploratory and Validation represent results for datasets 1 and 2, respectively.

**
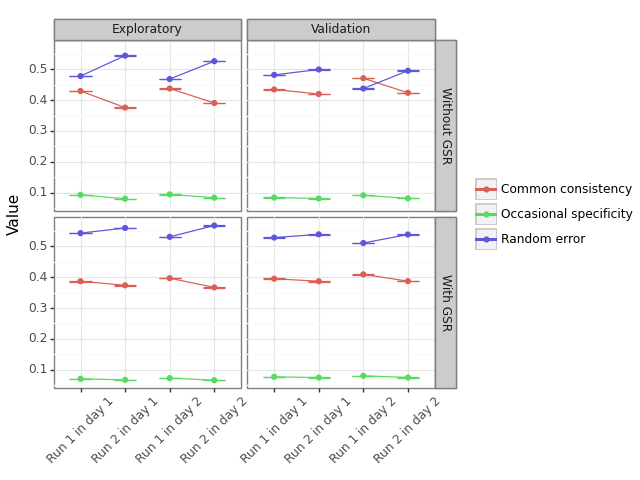
**

**Supplementary Figure S6:** Means and 95% confidence intervals of common consistency, occasional specificity, and random error in the four indicators of RSFC, calculated through multistate single-trait models without method factors. The criteria for model selection were applied. Results on the individuals with repeated measurements of RSFC in the counterbalanced order of phase encoding directions are shown (*N* = 199 in each dataset). Exploratory and Validation represent results for datasets 1 and 2, respectively.


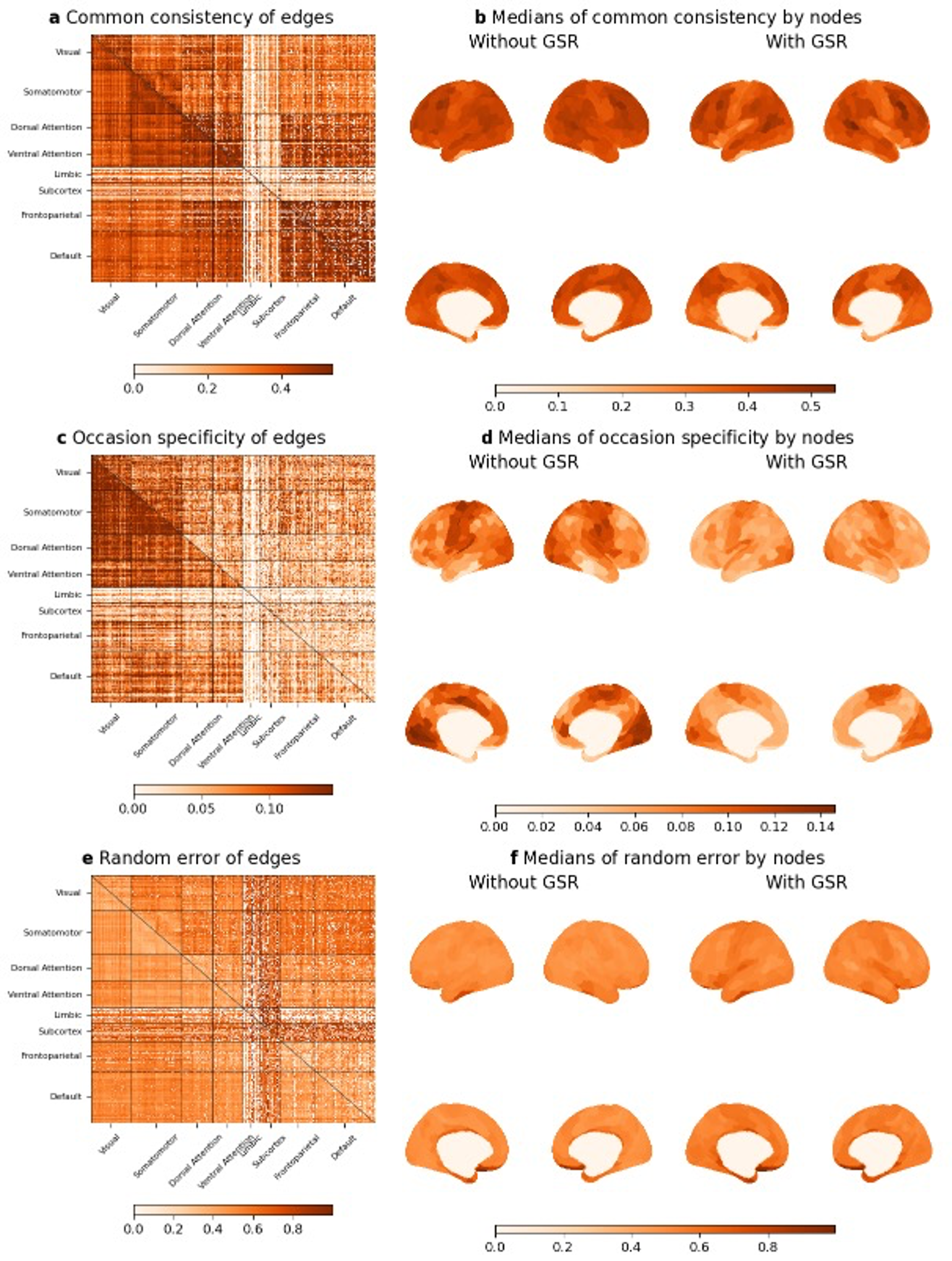


**Supplementary Figure S7:** Variance decompositions of the RSFC in subjects with counterbalanced measurements of phase encoding directions and scan orders (*N* = 199). The common consistency, occasional specificity, and random error of edges (**a**, **c**, **e**) and their medians by nodes (**b**, **d**, **f**) are shown. Common consistency, occasional specificity, and random error of edges were calculated by averaging values of four indicators in the measurement models. Model selection was not applied (see ‘Model evaluation’ in the Methods section). Heatmaps above diagonals represent results of GSR and those below diagonal results of no-GSR.


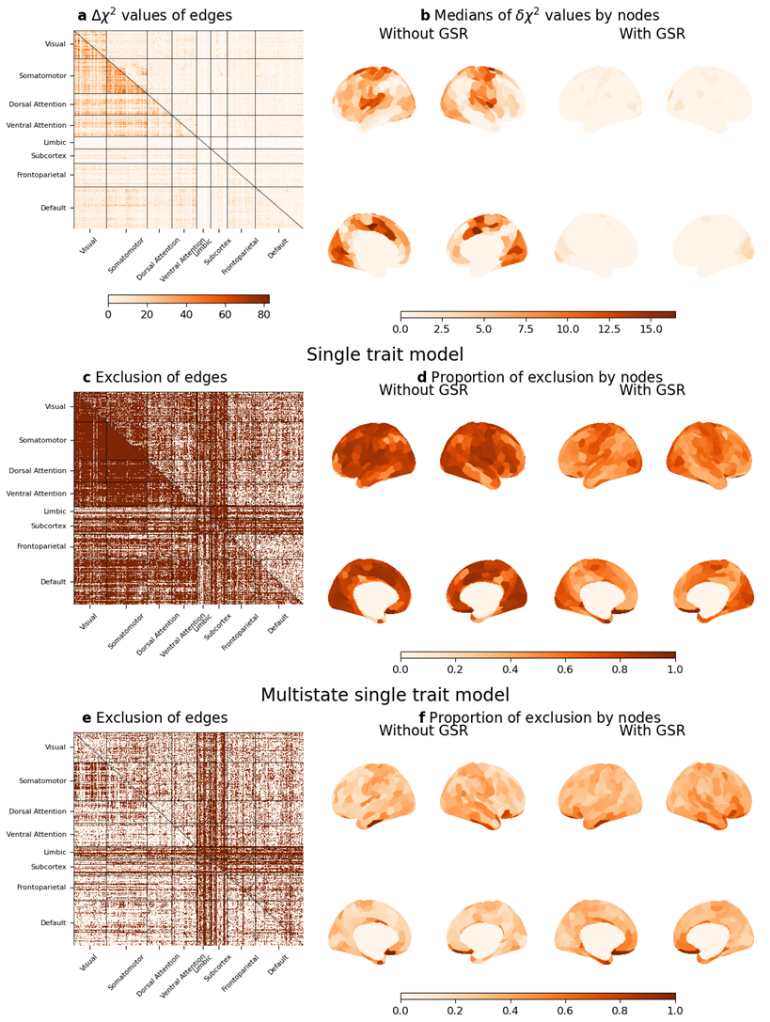


**Supplementary Figure S8:** Comparisons between measurement models of RSFC without covariates in dataset 2 (*N* = 204). **a**, **b**, Comparisons between single-trait model and multistate single-trait model. **a**, Heatmap of chi-square difference (*Δχ*^2^) values of edges. **b**, Heatmaps of medians of chi-square difference values of edges by nodes. **c**, d, Results of single-trait model. **e**, **f**, Results of multistate single-trait model. **g**, **h**, Results of multistate single-trait model with method effects of measurement order in days. **c**, **e**, **g**, Heatmaps of removed edges, where brown represents removed edge. **d**, **f**, **h**, Heatmaps of proportions of removed edges per nodes. Heatmaps above diagonals represent results of GSR and those below diagonal results of no-GSR. The criteria of model selection were not applied (see ‘Model evaluation’ in the Methods section).


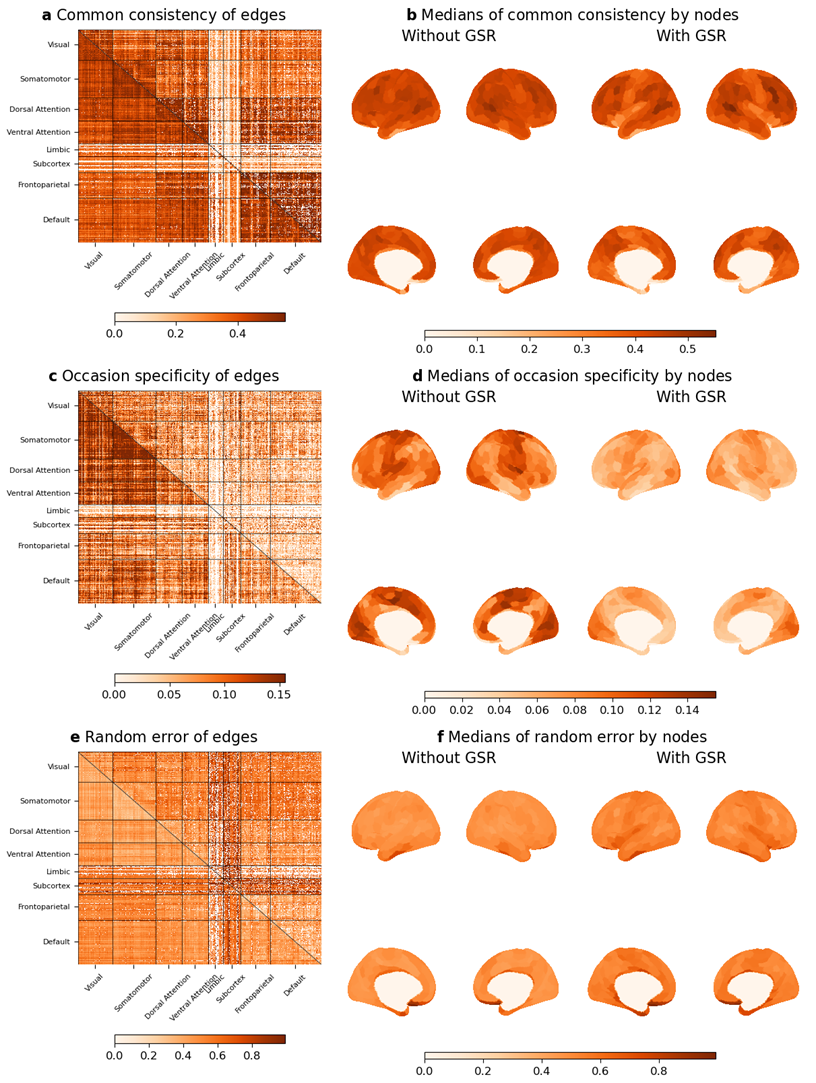


**Supplementary Figure S9:** Variance decompositions of RSFC in dataset 2 (*N* = 204). Common consistency, occasional specificity, and random error of edges (**a**, **c**, **e**) and their medians by nodes (**b**, **d**, **f**). Common consistency, occasional specificity, and random error of edges were calculated by averaging values of four indicators in the measurement models. Model selection was not applied (see ‘Model evaluation’ in the Methods section). Heatmaps above diagonals represent results of GSR and those below diagonal results of no-GSR.


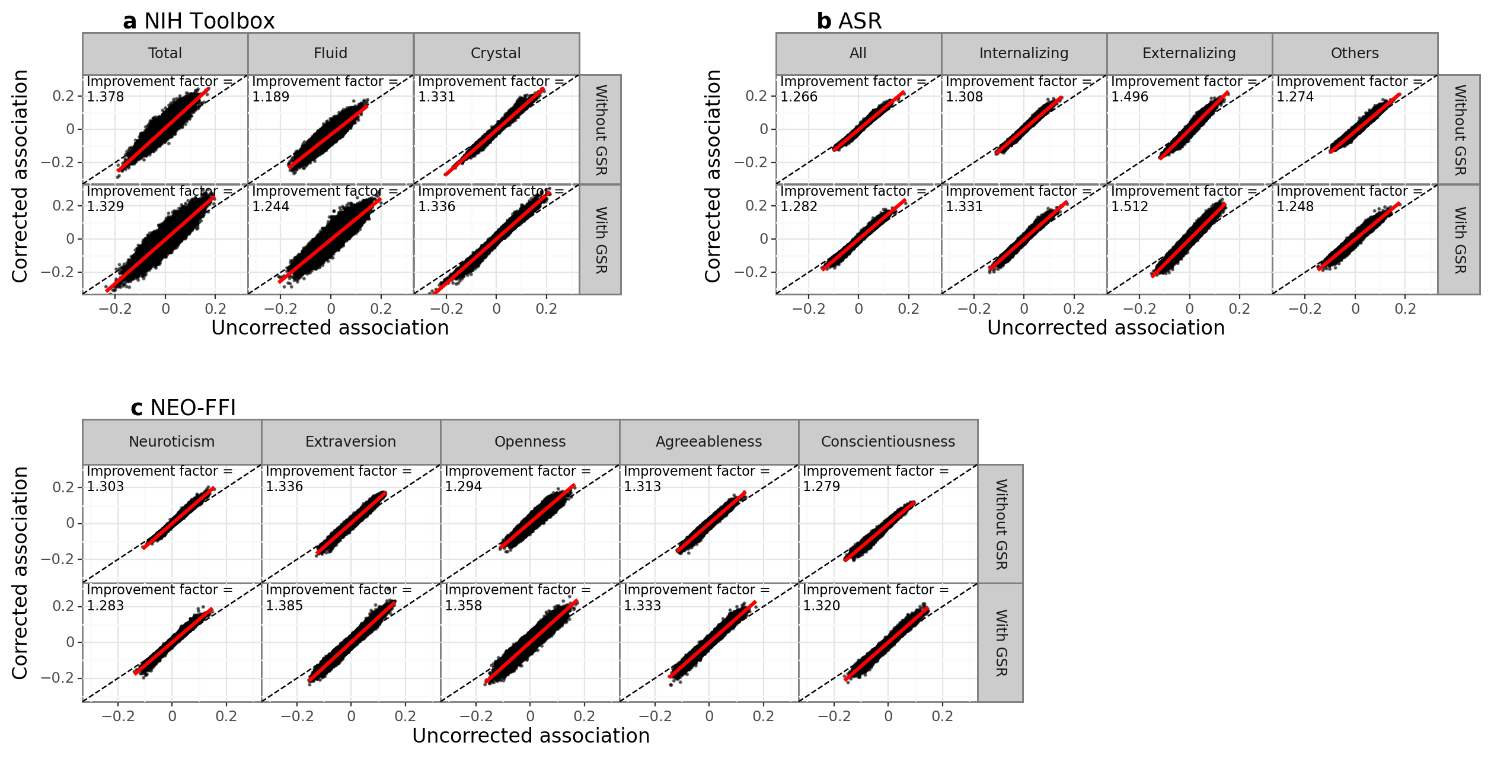


**Supplementary Figure S10:** Scatterplots of uncorrected and corrected RSFC-phenotype associations in each phenotype after edge selection. **a** NIH toolbox, **b** ASR, and **c** NEO-FFI. Uncorrected associations represent correlation between average scores of RSFC and those of items or tests of phenotype measures and corrected associations represent correlation between latent RSFC and latent phenotypes estimated in SEM.


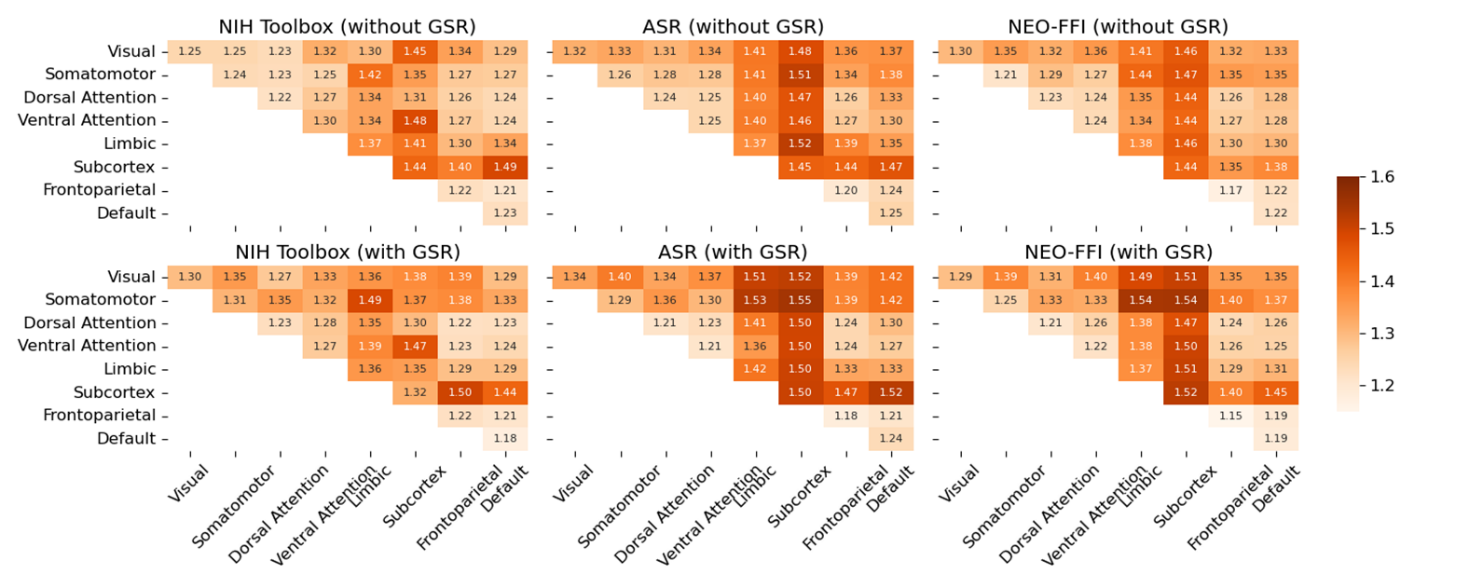


**Supplementary Figure S11:** Means of improvement factors in each phenotype in no-GSR and GSR conditions. Improvement factors represent linear regression slope of corrected association estimated in SEM on uncorrected association estimated in analyses on average scores of RSFC and phenotypes.


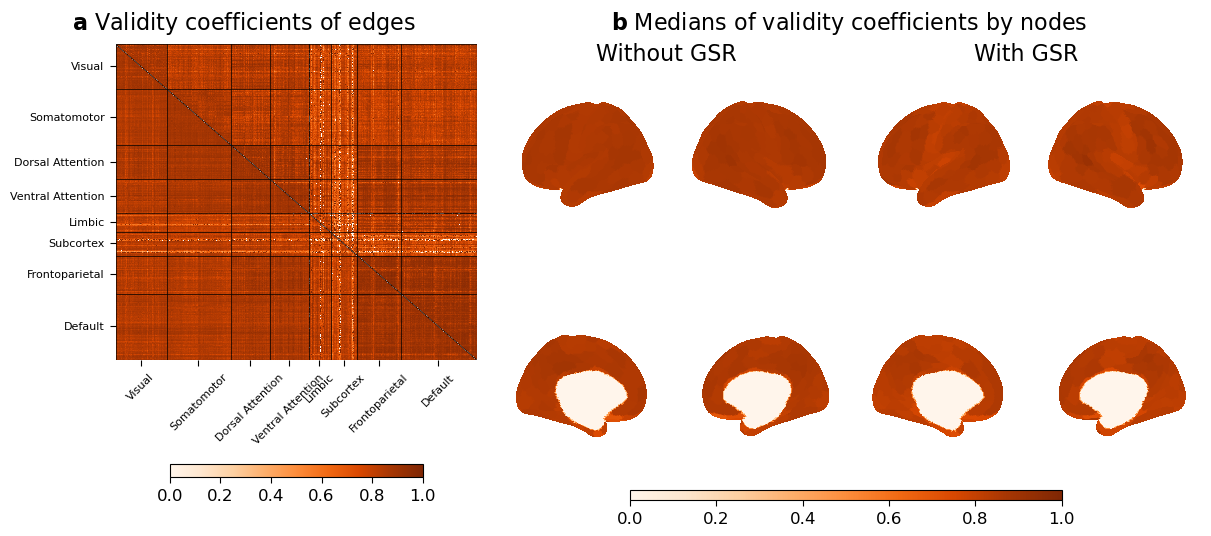


**Supplementary Figure S12:** Validity coefficients of factor score estimates of RSFC. Validity coefficients in edges (**a**) and their medians by nodes (**b**) in dataset 1 (*N* = 203). Validity coefficients represent correlation between factor score estimates and corresponding factors. Values > .80 are recommended.


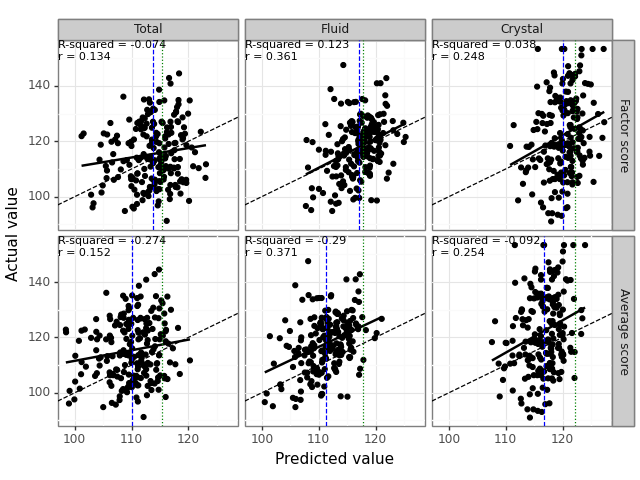


**Supplementary Figure S13:** An example of spurious performance of correlation coefficient as a performance metric (results on the NIH toolbox). Blue dashed and greed dotted lines represent averages of predicted and actual values, respectively. Although the means of predicted values did not correspond to those of actual values when using average scores of RSFC, correlation coefficients did not reflect this discrepancy.


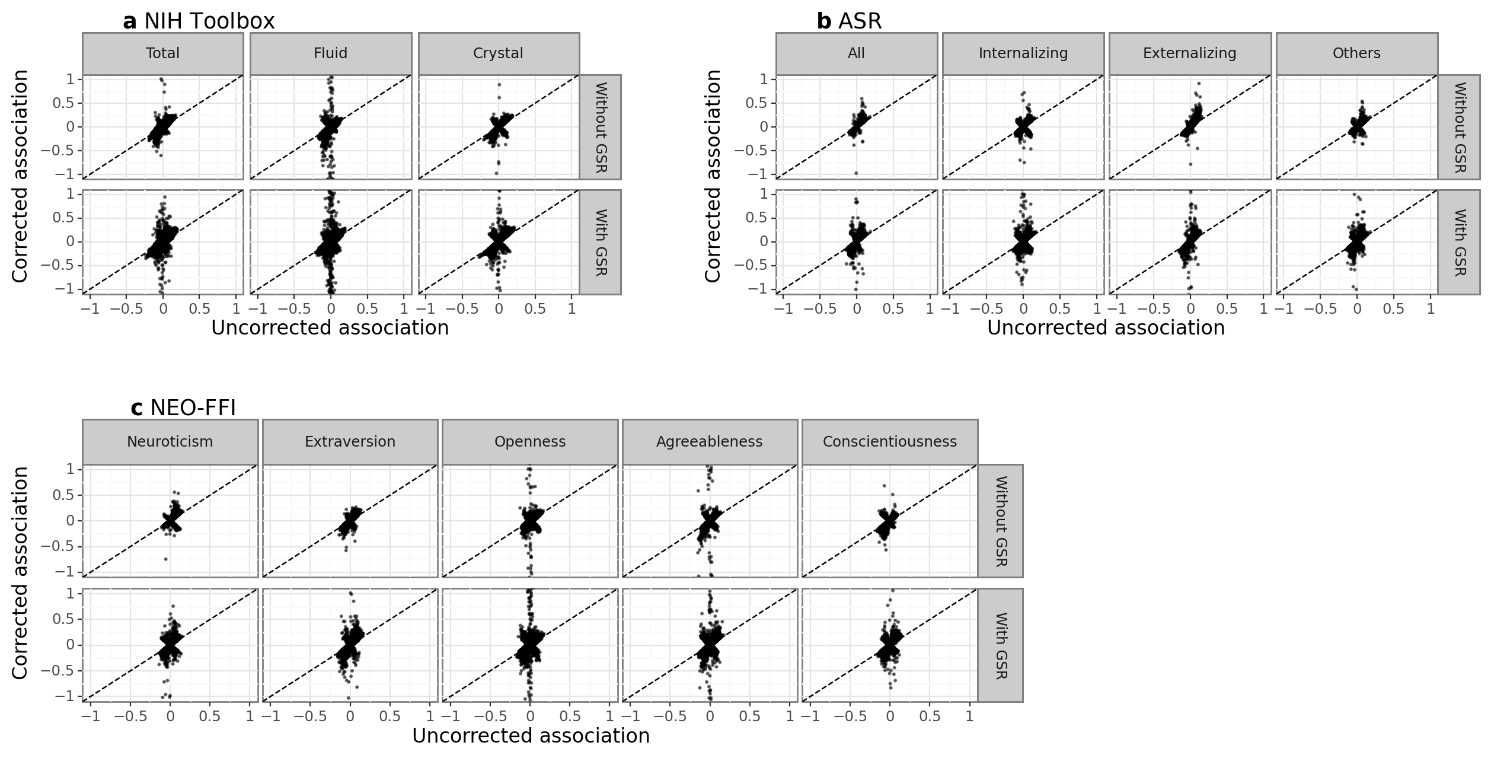


**Supplementary Figure S14:** Scatterplots of uncorrected and corrected RSFC-phenotype associations in each phenotype using full sample (*N* = 861) before edge selection. **a** NIH toolbox. **b** ASR. **c** NEO-FFI. See ‘Model evaluation’ in the Methods section for edge selection. Range of y-axis is limited from 0 to 1.

| a NIH Toolbox | | | | |
| --- | --- | --- | --- | --- |
| Total | Fluid | Crystal |  |  |
| 0.919 (0.904) | 0.889 (0.876) | 0.915 (0.896) |  |  |
| b ASR | | | | |
| All | Internalizing | Externalizing | Others |  |
| 0.950 (0.964) | 0.890 (0.909) | 0.831 (0.900) | 0.924 (0.943) |  |
| c NEO-FFI | | | | |
| Neuroticism | Extraversion | Openness | Agreeableness | Conscientiousness |
| 0.925 (0.939) | 0.908 (0.914) | 0.903 (0.905) | 0.900 (0.885) | 0.915 (0.920) |

**Supplementary Table S1:** Validity coefficients of factor score estimates of phenotypes in exploratory (out of parentheses) and validation (within parentheses) datasets. Validity coefficients represent correlation between factor score estimates and corresponding factors. Values > .80 are recommended.
